# Development of a novel glucose‐dendrimer based therapeutic targeting hyperexcitable neurons in neurological disorders

**DOI:** 10.1002/btm2.10655

**Published:** 2024-03-26

**Authors:** Anjali Sharma, Nirnath Sah, Rishi Sharma, Preeti Vyas, Wathsala Liyanage, Sujatha Kannan, Rangaramanujam M. Kannan

**Affiliations:** ^1^ Center for Nanomedicine at the Wilmer Eye Institute Johns Hopkins University School of Medicine Baltimore Maryland USA; ^2^ Anesthesiology and Critical Care Medicine Johns Hopkins University School of Medicine Baltimore Maryland USA; ^3^ Present address: Department of Chemistry Washington State University Pullman Washington USA

**Keywords:** brain injury, dendrimer, glucose dendrimer‐valproic acid conjugate, nanoparticle, seizure

## Abstract

Neuronal hyperexcitability and excitotoxicity lies at the core of debilitating brain disorders such as epilepsy and traumatic brain injury, culminating in neuronal death and compromised brain function. Overcoming this challenge requires a unique approach that selectively restores normal neuronal activity and rescues neurons from impending damage. However, delivering drugs selectively to hyperexcitable neurons has been a challenge, even upon local administration. Here, we demonstrate the remarkable ability of a novel, scalable, generation‐two glucose‐dendrimer (GD2) made primarily of glucose and ethylene glycol building blocks, to specifically target hyperexcitable neurons in primary culture, ex vivo acute brain slices, and in vivo mouse models of acute seizures. Pharmacology experiments in ex vivo brain slices suggest GD2 uptake in neurons is mediated through glucose transporters (GLUT and SGLT). Inspired by these findings, we conjugated GD2 with a potent anti‐epileptic drug, valproic acid (GD2–VPA), for efficacy studies in the pilocarpine‐mouse model of seizure. When delivered intranasally, GD2–VPA significantly decreased the seizure‐severity. In summary, our findings demonstrate the unique selectivity of glucose dendrimers in targeting hyperexcitable neurons, even upon intranasal delivery, laying the foundation for neuron‐specific therapies for the precise protection and restoration of neuronal function, for targeted neuroprotection.

**Translation Impact Statement**

Delivering drugs selectively to hyperexcitable neurons is a challenge. Herein, we demonstrate the remarkable ability of a novel glucose‐dendrimer, specifically targeting hyperexcitable neurons in mouse models of acute seizures. Valproic acid, an anti‐seizure drug with significant side effects, when conjugated to GD and delivered intranasally decreased severity of seizures acutely in the pilocarpine induced seizure model in mice. The unique neuronal uptake of glucose dendrimers and significant efficacy in a clinically relevant seizure model opens new avenues for translation of this therapy in status epilepticus.

## 1 INTRODUCTION

Brain injury can result in neuronal hyperexcitability that can contribute to several neurologic conditions like epilepsy, chronic pain, and Parkinson's disease.^1^, ^2^ Selective therapeutic targeting of these hyperexcitable neurons, can not only rescue neurons from excitotoxic death, but also limit disease propagation. However, selective targeting of neurons remains elusive, primarily because of the blood–brain barrier (BBB) and the diverse populations of neurons whose properties and functions vary across brain regions both in the normal brain and as a response to injury. Systemically administered drugs must get across the BBB, diffuse freely in the brain tissue and be selectively taken up by the target cells. This is a major challenge, with many ligand, antibody, viral vector‐based approaches explored, with mixed results.^3^, ^4^, ^5^, ^6^ Even if therapeutic drugs manage to cross the BBB, targeting neurons selectively remains a significant challenge and must be carefully considered as it can result in undesirable side effects including neurotoxicity and potential long‐term effects to brain functions leading to symptoms such as increased somnolence, dizziness, cognitive dysfunction, and so forth. These side effects are commonly seen with anti‐epileptic drugs that have good BBB penetration but have significant non‐specific neurological side effects.^7^, ^8^, ^9^ Since neurons function in a complex and diverse fashion in different brain regions, with regionally different overexpression of receptors, targeting subtypes of neurons in different regions of brain using a ligand‐based strategy may be impractical for broad applications.^5^, ^10^

Among myriads of differences, one common characteristic in most brain injury is higher metabolic activity and demand for glucose in neurons, at least in the acute phase of injury.^11^, ^12^, ^13^ For instance, both in vitro and in vivo seizure models consistently demonstrate heightened neuronal excitability, characterized by elevated firing frequencies, bursts of activity, and coordinated discharges.^14^, ^15^ This hyperexcitability necessitates higher glucose metabolism to adequately meet the substantial energy demands associated with increased neuronal firing rates, synaptic neurotransmission, ion channel function, and maintenance of membrane potentials.^16^, ^17^ Glucose is the primary metabolic source for brain^18^ and is transported across the BBB and made available to neurons and glia via specific glucose transporters.^19^, ^20^, ^21^, ^22^ In the presence of an excitotoxic injury, such as that seen with epilepsy, there is increased glucose transport into the affected neurons due to increased metabolic demand.^13^ Glut3 and SGLT transporters expressed by neurons can transport glucose from the interstitium and generate ATP through glycolysis, pentose phosphate pathway, and oxidative metabolism depending on the extent of neuronal stimulation.^20^, ^23^, ^24^, ^25^, ^26^

Here we present the development of a novel non‐biodegradable glucose dendrimer (GD2) platform synthesized by copper catalyzed click chemistry approach that will exploit the increased glucose demand and uptake of hyperexcitable neurons to specifically target and deliver anti‐epileptic drugs to these cells, in an animal model of epilepsy without providing metabolizable glucose. Roughly two decades ago, the process of creating dendrimers was seen as a time‐consuming endeavor. The synthesis of even the most widely explored polyamidoamine (PAMAM) dendrimer requires ~8 synthetic steps and large excess of reagents needed to produce a flawless dendrimer. However, the advent of click chemistry in the realm of nanotechnology has revolutionized this landscape. We can now develop highly complex macromolecular structures with ease and can harness the physiochemical properties of a macromolecule in a highly controlled manner for desired biological applications.^27^, ^28^

## 2 MATERIALS AND METHODS

### 2.1 Chemistry: Materials

β‐D‐(+)‐Glucose pentaacetate (≥99%), boron trifluoride diethyl etherate, sodium azide, copper sulfate pentahydrate, sodium ascorbate, sodium methoxide, 4‐dimethylaminopyridine (DMAP), *N*‐ethyl‐N′‐(3‐dimethylaminopropyl)carbodiimide (EDC), and tetraethyleneglycol were purchased from Sigma‐Aldrich US and used as received. Azido‐PEG4‐alcohol was purchased from BroadPharm. Cy5 NHS ester was purchased from GE healthcare and used as received. Valproic acid is purchased from ApexBio (Boston, MA, USA). All other ACS grade solvents were used as received. Anhydrous *N*,*N*‐dimethyl formamide, dichloromethane, and methanol were received from Sigma. Deuterated solvents *N*,*N*‐dimethylsulfoxide (DMSO‐*d6*), water (D_2_O), and chloroform (CDCl_3_) were received from Sigma‐Aldrich (St Louis, MO, USA). All the reactions in the organic medium were performed in standard oven‐dried glassware under an inert nitrogen atmosphere unless otherwise stated. Dialysis membrane (MWCO 1 kDa) was purchased from Spectrum Laboratories Inc.

### 2.2 Characterization

#### 2.2.1 Nuclear magnetic resonance

The nuclear magnetic resonance (NMR) spectra were recorded on a Bruker 500 MHz spectrometer at ambient temperatures. The chemical shifts of the residual protic solvent of CDCl_3_ (^1^H, *δ* 7.27 ppm); D_2_O (^1^H, *δ* 4.7 ppm), and DMSO‐*d6* (^1^H, *δ* 2.50 ppm) were used for chemical shifts calibration. The chemical shifts are reported in ppm and are relative to an internal standard (tetramethylsilane).

#### 2.2.2 Mass spectroscopy

Matrix assisted laser desorption ionization time of flight (MALDI‐TOF) experiments were performed on Bruker Autoflex MALDI‐TOF instrument. The compound was dissolved in ultra‐purified water at 5 mg/mL. The matrix was dissolved in 50:50 (v/v) acetonitrile:water mixture at 10 mg/mL concentration. For the compounds with molecular weights <10k, the reflectron mode was used with 2,5‐dihydroxybenzoic acid (DHB) as the matrix. For the other larger compounds, the linear mode was used with sinapinic acid as the matrix. The preparation of the samples was performed by mixing 10 μL of the compound solution with 10 μL of the matrix solution after which 3 μL of the sample was spotted on a MALDI plate. Laser power used for this purpose was 55%–100%.

#### 2.2.3 High performance liquid chromatography

The purity of the intermediates and the final dendrimers was analyzed using high performance liquid chromatography (HPLC). The HPLC instrument (Waters Corporation, Milford, MA) is equipped with a 1525 binary pump along with an In‐Line degasser AF and a 717 plus auto sampler. The instrument has a 2998 photodiode array detector and a 2475 multi λ fluorescence detector interfaced with Waters Empower software. HPLC analyses were performed using C18 are symmetry 300, 5 μm, 4.6 × 250 mm from Waters. The chromatographs were recorded at 210 wavelengths. A gradient flow HPLC method was used starting with 100:0 (Solvent A: 0.1% TFA and 5% ACN in water; Solvent B: 0.1% TFA in ACN); gradually increasing to 25:75 (A:B) at 20 min, 10:90 (A:B) in 25 min and finally returning to 100:0 at 30 min maintaining a flow rate of 1 mL/min.

#### 2.2.4 Dynamic light scattering and zeta potential

The size distribution by number and the zeta potential distribution of the dendrimers were determined by using a Zetasizer Nano ZS (Malvern Instrument Ltd., Worchester, UK). The instrument is equipped with a 50 mW He–Ne laser (633 nm). The sample for the size measurement was prepared by dissolving the dendrimer in deionized water (18.2 Ω) to make a solution with a final concentration of 0.5 mg/mL. The sample solution was vortexed and sonicated for 2 min and then filtered through 0.2 μm syringe filters (Pall Corporation, 0.2 μm HT Tuffryn membrane) directly into the cell (UV transparent disposable cuvette, Dimensions: 12.5 × 12.5 × 45 mm, SARSTEDT) and the measurements were performed. The sample for zeta potential measurement was prepared at a concentration of 0.2 mg/mL in 10 mM NaCl. The sample was filtered through 0.2 μm syringe filters. The cell used was Malvern Zetasizer Nanoseries disposable folded capillary cell. Both the size and the zeta potential measurements were performed in triplicate.

### 2.3 Synthetic procedures

The synthesis of compounds (**1**), (**2**), and (**6**) was achieved using previously published protocols.^29^

#### 2.3.1 Synthesis of compound **3**

Hexapropargylated compound **1** (0.5 g, 1 mmol) and an azido derivative 2 (4.1 g, 7.4 mmol; 1.2 equiv. per acetylene) were suspended in a 1:1 mixture of DMF and water in a 50 mL round bottom flask. To this were added CuSO_4_·5H_2_O (5 mol%/acetylene, 75 mg) and sodium ascorbate (5 mol%/acetylene, 60 mg) dissolved in the minimum amount of water. The reaction was kept at 50°C for 6 h. The reaction mixture was dialyzed against DMF followed by water dialysis containing EDTA. The EDTA was further removed by extensive water dialysis. The product was lyophilized to obtain compound **3**.

^1^H NMR (500 MHz, CDCl_3_) *δ* 8.07 (s, 6H), 5.20 (t, *J* = 9.1 Hz, 6H), 5.06 (dd, *J* = 21.7, 12.2 Hz, 6H), 4.98 (t, *J* = 8.7 Hz, 6H), 4.71–4.54 (m, 30H), 4.32–4.20 (m, 6H), 4.18–4.1 (m, 6H), 4.0–3.88 (m, 18H), 3.76–3.56 (m, 72H), 3.44 (s, 12H), 3.30 (s, 4H), 2.11–1.98 (dd, *J* = 23.3, 16.3 Hz, 72H).

^13^C NMR (126 MHz, CDCl_3_) *δ* 170.6, 170.2, 169.4, 145.0, 123.6, 100.8, 72.8, 71.7, 71.2, 70.6, 70.5, 70.4, 70.2, 69.4, 69.2, 68.3, 61.9, 50.0, 29.6, and 20.6.

MALDI‐ToF: m/z: calculated for C_160_H_244_N_18_O_85_: 3777.53, found: 3800.73 [M + Na]^+^.

#### 2.3.2 Synthesis of compound **4**

The peracetylated generation 1 glucose dendrimer (**3**, 1 g, 0.26 mmol) was dissolved in anhydrous methanol and sodium methoxide was added to adjust the pH around 8.5–9. The reaction was stirred overnight at room temperature, then diluted with methanol and pH was adjusted with Amberlist IR‐120+ around 6–7. The reaction mixture was separated by filtration and the solvent removed by rotary evaporation.

^1^H NMR (500 MHz, D_2_O) *δ* 8.00–7.85 (m, 6H), 4.49 (dd, *J* = 11.6, 6.5 Hz, 12H), 4.44–4.40 (m, 10H), 4.38 (d, *J* = 7.9 Hz, 6H), 3.99–3.91 (m, 6H), 3.90–3.81 (m, 14H), 3.80 (d, *J* = 2.0 Hz, 4H), 3.75–3.66 (m, 6H), 3.68–3.58 (m, 18H), 3.59–3.45 (m, 48H), 3.44–3.25 (m, 26H), 3.23–3.09 (m, 12H).

MALDI‐ToF: m/z: calculated for C_112_H_196_N_18_O_61_: 2769.28, found: 2791.85 [M + Na]^+^.

#### 2.3.3 Synthesis of compound **5**

Compound **4** (2 g, 0.721 mmol) was dissolved in anhydrous dimethylformamide (DMF, 50 mL) by sonication. To this stirring solution, sodium hydride (60% dispersion in mineral oil, 951 mg, 39.65 mmol) was slowly added in portions at 0°C. The solution was additionally stirred for 15 min at 0°C. This was followed by the addition of propargyl bromide (3.85 mL, 34.608 mmol, 80% (w/w) solution in toluene) at 0°C and the stirring was continued at room temperature for another 6 h. The reaction mixture was quenched with ice and water, filtered, and dialyzed against DMF, followed by the water dialysis to afford compound **5**.

^1^H NMR (400 MHz, CDCl_3_) *δ* 8.0–7.92 (m, 6H), 4.62–3.39 (m, 214H), 2.47 (dt, *J* = 7.0, 2.3 Hz, 24H).

MALDI‐TOF: m/z: calculated for C_184_H_244_N_18_O_61_: 3681.65, found: 3721.19 [M + K]^+^.

#### 2.3.4 Synthesis of compound **7**

Compound **5** (1 g, 0.54 mmol) and compound **6** (5.37 g, 14.11 mmol) were suspended in a 1:1 mixture of DMF and water in a 50 mL round bottom flask equipped with a magnetic stir bar. To this CuSO_4_·5H_2_O (5 mol%/acetylene, 5 mg) and sodium ascorbate (5 mol%/acetylene, 10 mg) dissolved in the minimum amount of water were added. The reaction was heated at 50°C for 8 h. Upon completion, the reaction mixture was dialyzed against DMF followed by water dialysis containing EDTA. The EDTA was further removed by extensive water dialysis. The product was lyophilized to obtain compound **7**.

^1^H NMR (500 MHz, D_2_O) *δ* 8.09–7.79 (m, triazole H), 4.92–4.78 (m, dendrimer H), 4.66–4.32 (m, dendrimer H), 4.04–3.89 (m, dendrimer H), 3.88–3.66 (m, dendrimer H), 3.66–3.33 (m, dendrimer H), 3.31–3.07 (m, dendrimer H).

MALDI‐TOF: m/z: calculated for C_520_H_892_N_90_O_277_: 12,830, found: 12,863.

HPLC: 99% purity, retention time: 7.9 min.

#### 2.3.5 Synthesis of compound **8**

Compound **7** (1 g, 0.076 mmol) was dissolved in anhydrous dimethylformamide (DMF, 50 mL) by sonication. To this stirring solution, sodium hydride (60% dispersion in mineral oil, 36 mg, 1.5 mmol) was slowly added at 0°C. The solution was additionally stirred for 15 min at 0°C. This was followed by the addition of propargyl bromide (0.06 mL, 0.456 mmol, 80% (w/w) solution in toluene) at 0°C and the stirring was continued at room temperature for another 6 h. The reaction mixture was quenched with ice and water, filtered, and dialyzed against DMF, followed by the water dialysis to afford compound **8**.

^1^H NMR (500 MHz, D_2_O) *δ* 8.15–8.0 (m, triazole H), 5.0–4.9 (m, dendrimer H), 4.82 (m, dendrimer H), 4.70–4.53 (m, dendrimer H), 4.51–4.37 (m, dendrimer H), 4.15–3.92 (m, dendrimer H), 3.89 (m, dendrimer H), 3.92–3.74 (m, dendrimer H), 3.74–3.35 (m, dendrimer H), 3.29 (m, dendrimer H) 2.50 (m, acetylene H).

#### 2.3.6 Synthesis of compound **9**

Compound **8** (0.1 g, 0.0076 mmol) and Cy5‐azide (8.7 mg, 0.0084 mmol) were suspended in a 1:1 mixture of DMF and water in a 50 mL round bottom flask equipped with a magnetic stir bar. To this CuSO_4_·5H_2_O (5 mol%/acetylene, 5 mg) and sodium ascorbate (5 mol%/acetylene, 10 mg) dissolved in the minimum amount of water were added. Upon completion, the reaction mixture was dialyzed against DMF followed by water dialysis containing EDTA. The EDTA was further removed by extensive water dialysis. The product was lyophilized to obtain compound **9**.

^1^H NMR (500 MHz, D_2_O) *δ* 8.2–7.2 (m, triazole H), 6.6–6.0 (m, CY5 H), 5.0–2.9 (m, dendrimer H), 2.2–1.1 (m, Cy5 H).

HPLC: Purity >99%; retention time: 7.79 min.

#### 2.3.7 Synthesis of compound **11**

To a stirring solution of VPA (300 mg, 2.08 mmol) in anhydrous dichloromethane (DCM; 5 mL), EDC (478.5 mg, 2.49 mmol) and DMAP (127.1 mg, 1.04 mmol) were added. The solution was stirred at room temperature for 30 min followed by the addition of a solution of azido‐PEG4‐alcohol (547.2 mg, 2.49 mmol) in DCM (1 mL). The reaction mixture was stirred under nitrogen atmosphere at room temperature for 12 h. The completion of the reaction was monitored via TLC. Upon completion, the reaction mixture was diluted with DCM (50 mL) and washed with water (50 mL ×3). The organic layer was then washed with brine and dried over sodium sulfate. The dried organic layer was evaporated under vacuo to afford VPA‐azide.

^1^H NMR (500 MHz, CDCl_3_) *δ* 4.30–4.18 (m, 2H), 3.75–3.59 (m, 12H), 3.39 (t, *J* = 5.1 Hz, 2H), 2.39 (tt, *J* = 9.0, 5.3 Hz, 1H), 1.59 (dtd, *J* = 13.2, 8.7, 6.6 Hz, 2H), 1.49–1.35 (m, 2H), 1.34–1.20 (m, 4H), 0.89 (t, *J* = 7.3 Hz, 6H).

#### 2.3.8 Synthesis of compound **12**

To a stirring solution of GD2 (200 mg, 0.016 mmol) and 5‐hexynoic acid (17.5 mg, 0.155 mmol) in anhydrous *N*,*N* dimethylformamide (DMF, 5 mL), EDC (35.7 mg, 0.186 mmol) and DMAP (9.5 mg, 0.078 mmol) were added. The solution was stirred at room temperature for 24 h. The solution was then diluted with DMF (100 mL) and dialyzed against DMF for 12 h. The DMF was changed every 4 h to remove organic impurities. The DMF was then removed by dialyzing against water for 12 h, changing water every 2–3 h. The solution was then lyophilized to afford GD2‐acetylene as white solid.

^1^H NMR (500 MHz, D_2_O) *δ* 8.10 (m, *GD2 internal triazole H*), 8.05–7.96 (m, *GD2 triazole H*), 4.99 (*GD2 H*), 4.75–4.38 (m, *GD2 H*), 4.14–3.17 (m, *GD2 H*), 2.60–2.45 (m, *linker H*), 2.28–2.16 (m, *linker H*), 1.84–1.69 (m, *linker H*).

#### 2.3.9 Synthesis of compound **13**

To a stirring solution of GD2‐acetylene (100 mg, 0.0074 mmol) and VPA‐azide (20.5 mg, 0.059 mmol) in DMF/H_2_O (1:1; 5 mL), a catalytic amount of copper‐sulfate pentahydrate and sodium ascorbate was added. The reaction was stirred at room temperature for 12 h. The reaction mixture was then diluted with DMF (100 mL) and dialyzed against DMF for 12 h. The DMF was changed every 4 h to remove organic impurities. The DMF was then removed by dialyzing against water for 12 h, changing water every 2–3 h. The solution was then lyophilized to afford GD2–VPA as an off‐white solid.

^1^H NMR (500 MHz, DMSO) *δ* 8.16–7.95 (m, *triazole H*), 7.83 (s, *triazole H from VPA conjugation*), 5.24–4.67 (m, *GD2 H*), 4.63–4.29 (m, *GD2 H*), 4.25–4.01 (m, *GD2 H and PEG H*), 3.97–2.76 (m, *GD2 H and PEG H*), 2.70–2.59 (m, linker *H*), 2.42–2.26 (m, *linker H and VPA H*), 1.92–1.76 (m, *linker H*), 1.71 (m, *linker H*), 1.48 (dt, *VPA H*), 1.38 (p, *VPA H*), 1.31–1.14 (m, *VPA H*), 0.84 (t, *VPA H*).

HPLC: Purity: 99%; retention time: 11.93 min.

### 2.4 In vitro release study of GD2–VPA conjugates

In vitro release study was performed at plasma conditions at pH 7.4 and intracellular endosomal compartment conditions at pH 5.5 (citrate buffer plus esterase). GD2–VPA solutions were prepared in PBS and citrate buffer containing esterase and were kept at 37°C to mimic physiological conditions. Release samples were taken out periodically at given time points. A standard curve for VPA was created using HPLC at 210 nm. The amount and percent VPA release from GD2–VPA samples were calculated from area under the curve using the standard curve for free VPA.

### 2.5 In vitro and in vivo studies

*Mice*: C57BL/6 Thy1‐YFP (Strain #003709) and wild type C57BL/6 (Strain #000664). Six to 8 weeks old male and female mice (20–30 g) were purchased from Jackson and were subsequently bred and housed in the animal facility with a 12‐h light and 12‐h dark cycle under the controlled temperature and humidity (temperature: 21 ± 0.5°C; humidity: 55% ± 5%). Standard rodent feed was provided ad libitum. All the in vivo dendrimer localization and efficacy studies were carried out according to protocol no. M021M208 (approved on August 2, 2021) approved by the Johns Hopkins University Animal Care and Use Committee (IACUC). Animals were closely observed post‐pilocarpine induced seizures for signs of pain and suffering for humane end points, if required. The Thy1‐YFP mice were also used for acute brain slice experiments. Wild type C57BL/6 mice (males and females) were used for pilocarpine‐induced seizure studies as described previously.^30^ Scopolamine methyl nitrate was injected intraperitoneally (2 mg/kg, Sigma‐Aldrich S2250), followed by pilocarpine hydrochloride (i.p.; Sigma‐Aldrich P6503 at 280–300 mg/kg) after 30 min. Behavioral seizures were monitored and scored using modified Racine's scale.^30^

#### 2.5.1 Isolation and culture of primary neurons from rabbit kit brain tissue

Primary hippocampal neuron cultures were used in this study using standard procedure. Briefly, brain hippocampi were micro dissected from postnatal day 1 rabbit kit followed by removing blood vessels and meninges in ice‐cold dissection solution containing 1× Hank's Balanced Salt solution, 1× penicillin/streptomycin, 1 mM sodium pyruvate, 10 mM HEPES, and 30 mM glucose. Subsequently, hippocampii were chopped and digested using the Papain Dissociation kit according to the manufacturer's protocol (Cat # LK003160, Worthington, USA). The digested tissue in the buffer was triturated with a sterile fire‐polished glass pipette to dissociate tissue clumps and cells and then centrifuged at 4°C for 5 min at 300*g*. The pellet was resuspended in Earle's Balanced Salt Solution (EBSS) containing ovomucoid protease inhibitor with bovine serum albumin and deoxyribonuclease. Next, a discontinuous density gradient was prepared by pipetting the cell suspension onto a 5 mL layer of albumin‐ovomucoid inhibitor solution and centrifuged at 70*g* for 6 min at room temperature to remove the supernatant containing noncellular debris. The resulting cell pellet was resuspended in neurobasal medium supplemented with 1× GlutaMAX, 2% B27, 1% penicillin–streptomycin, and 1% heat‐inactivated horse serum. Fifty thousand cells were seeded on poly‐D‐lysine and laminin‐coated coverslips and incubated at 37°C. After 24 h, media was replaced with fresh media containing 5 μM cytosine arabinoside. Half of the culture medium was changed every week.

For immunocytochemistry, neuron cultures were washed with PBS and fixed with formalin for 10 min followed by another PBS wash. Next, the cultures were blocked with 10% donkey serum for 30 min and subsequently incubated overnight with anti‐beta III tubulin antibody (1:1000, Abcam, MA, USA). Upon washing the culture coverslips, they were incubated with alexa fluor conjugated secondary antibody (donkey anti‐rabbit AF488, 1:250) for 1 h. The coverslips were then treated with DAPI (1:5000) for 5 min and washed and mounted.

#### 2.5.2 Animal model of cerebral palsy

Time pregnant New Zealand white rabbits (Robinson Services Inc.) underwent laparotomy surgery at gestational day 28 according to the protocol no. RB22M148 approved on September 17, 2020, by the Johns Hopkins University Animal Care and Use Committee (IACUC). Briefly, during laparotomy, rabbits received a total of 1800 EU (endotoxin units) of LPS (*Escherichia coli* serotype O127:B8, Sigma‐Aldrich, St. Louis, MO) injection along the wall of the uterus.^31^ Rabbits were induced on G30 with intravenous injection of Pitocin (0.5 U/kg) (JHP Pharmaceuticals, Rochester, MI) and sacrificed for live brain slice experiments on day of birth.

#### 2.5.3 Behavioral seizures

For efficacy studies, the male and female mice (weighing at least 20 g) were randomly allocated to different experimental groups. We used a single dose of pilocarpine to induce the SE. Prior to seizure induction, scopolamine methyl nitrate was injected intraperitoneally to antagonize the peripheral effects of pilocarpine. The animals were video‐recorded in their individual cages and the convulsive activity was scored based on the modified Racine scale^32^, ^33^ as follows: Stage 0, normal activity; Stage 1, freezing and slight head nodding; Stage 3, head bobbing, wet dog shakes, straub tail; Stage 4, partial myoclonus, occasional jerks, body tremors, intensified freezing, or uncontrolled circling movement; Stage 4, increased immobility and freezing, uncontrolled circling movement; Stage 5, continuous straub tail, loss of limb control followed by generalized tonic–clonic seizures, oro‐alimentary automatism, or one episode of rearing; Stage 6, loss of balance, more than one episode of rearing followed by occasional falling, jumping and rolling over, generalized tonic extension of the body, cardiopulmonary collapse, and death. The continuous behavioral seizures were recorded for 180 min, and analysis was performed by blinded experimenter. Stages 1 and 2 were categorized as low grade, Stage 3 as medium grade, and stages 5 and 6 as high‐grade seizures. Each counted episode lasted for at least for 30 s, typically separated by the other with variable durations. Periods between stages 3 and 6 were considered as continuous low‐grade seizures as observed in these animals for 180 min. All the behavior experiments were carried out by the same experimenter in the same behavior room; the room was blocked at the time of behavior to control the effect of unknown environmental variables on the experimental design and data acquisition.

Our inclusion and exclusion criteria for the seizure experiment were based on the ILAE guidelines. All animals were recorded, and seizure scoring was carried out to screen for the onset of SE. We considered repeated and sufficiently prolonged continuous seizures of medium grade or higher with short intervals (at least one medium or high‐grade seizure within 5 min) as the development of SE. Here, we have only looked at the SE with prominent motor phenomenon including the bilateral tonic–clonic SE (convulsive SE) and focal, myoclonic, tonic, and hyperkinetic SE^34^, ^35^; therefore, we do not claim the quantification of the non‐convulsive or electrographic seizures in these animals.

### 2.6 Ex vivo brain slices preparation

CP rabbit kits or Thy1‐YFP mice were deeply anesthetized with isoflurane and decapitated. Brains were removed and transferred to oxygenated (95% O_2_/5% CO_2_), ice‐cold N‐methyl‐D‐glucamin (NMDG)‐based buffer (Buffer 1, in mM: 92 NMDG, 2.5 KCl, 10 MgSO_4_, 0.5 CaCl_2_, 1.2 NaH_2_PO_4_, 30 NaHCO_3_, 25 glucose, 20 HEPES, 5 sodium ascorbate, 3 sodium pyruvate, and 2 thiourea; pH 7.4). Coronal brain slices (300 μM) were then obtained using a vibratome (VT1200, Leica). Slices were first incubated in the NMDG‐based solution at 34°C for 10 min, then transferred and maintained in the same solution for an hour at room temperature before starting the subsequent experiment. Dendrimer‐Cy5 was added in 5 mL of Buffer 2 (in mM: 125 NaCl, 2.5 KCl, 1 MgCl_2_, 2 CaCl_2_, 1.25 NaH_2_PO_4_, 26 NaHCO_3_, and 20 glucose; pH 7.4) containing brain slices and incubated for 30 min while oxygenation at room temperature. Brain slices were fixed with formalin and immune‐stained for neuronal (MAP2 or PGP) proteins for visualized under an upright microscope equipped with laser scanning confocal optics (LSM 880, Zeiss).

### 2.7 Immunohistochemistry

For immunohistochemistry, animals were perfused with saline and post‐fixed in 10% formalin for 24 h followed by cryoprotection in 30% sucrose solution for 2 days. Coronal sections (30 μm, 1:6 series) were collected and blocked by 5% normal donkey serum in 0.1 M PBS. Subsequently, sections were then incubated overnight at 4°C with goat anti‐IBA1 (1:500, Abcam, MA. USA), followed by incubation with secondary antibodies for 2 h at room temperature. Upon incubation with DAPI (1:1000, Invitrogen) for 15 min, slides were washed, dried and cover slipped with mounting medium. Similarly, for acute CP brain slice immunostaining, slices were fixed overnight, washed and incubated with primary antibody anti‐PGP antibody (1:100, Abcam, MA, USA) or anti‐MAP2 antibody (1:100, Aves Lab, OR, USA) followed by 2‐h incubation in secondary antibody solution. Confocal images were acquired with Zeiss ZEN LSM 710 (Zeiss, CA, USA) and processed with ZEN software.

### 2.8 Statistics

We summarized the data using mean values ± SEM and used either *t*‐test or one way‐ANOVA for group comparisons. We used the Mann–Whitney *U* test was used to test the significance of seizure severity scores. Fischer exact test was used to analyze the significance of seizures in mice, Kruskal–Wallis test followed by Dunn's multiple comparisons test was used to analyze seizure latencies. Unless otherwise stated, the Bonferroni corrections were used to adjust for multiple comparisons. All the data analyses were performed using GraphPad Prism software (version 9.5.1). Statistical significance was set as *p* < 0.05, and all tests were two‐sided. Statistical techniques were not employed for predefining the sample size, though our sample sizes align with those reported in earlier literature.^36^, ^37^

## 3 RESULTS

### 3.1 Synthesis and characterization of glucose dendrimer

**FIGURE 1:**
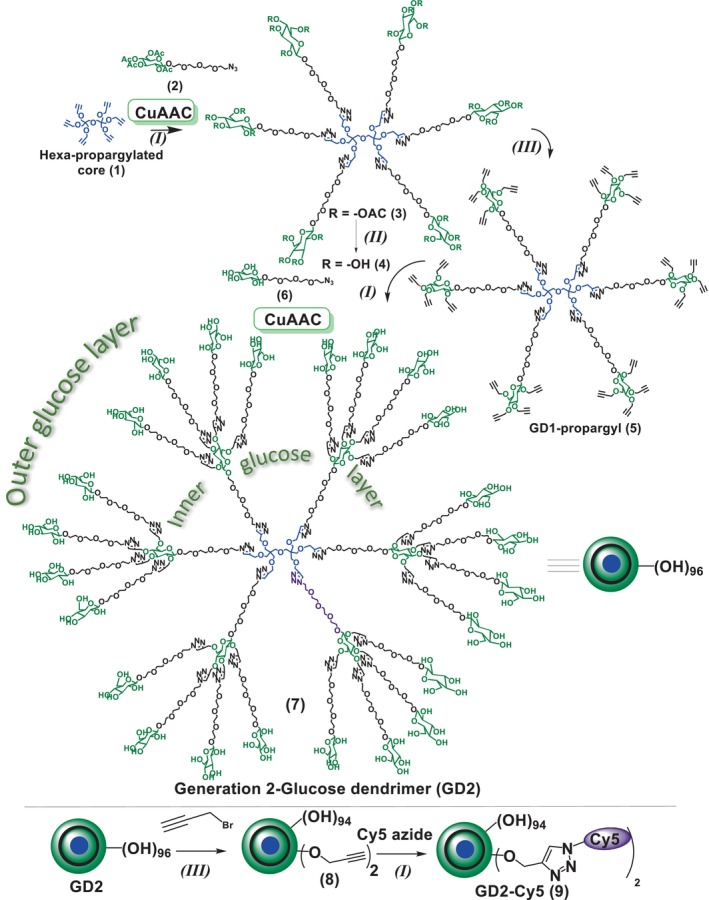
Synthetic sequence of GD2. Schematic representation of the stepwise synthesis of second‐generation glucose dendrimer (GD2) and its conjugate with the attachment of near infra‐red fluorophore Cy5. Reagents and conditions: (I) CuSO_4_·5H_2_O (5 mol%/acetylene), sodium ascorbate, DMF:water (1:1), RT, 6 h, 50°C; (II) anhydrous methanol, sodium methoxide, RT, 12 h; (III) anhydrous DMF, sodium hydride, 0°C, RT, 6 h.

**FIGURE 2:**
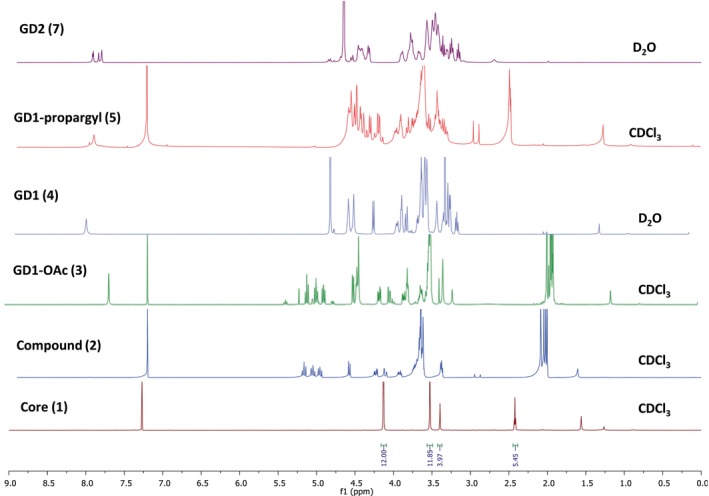
Structural elucidation of GD2 and intermediates via NMR. ^1^H NMR of GD2 and intermediate depicting the characteristic peaks of protons at each synthetic step from core to final dendrimer.

**FIGURE 3:**
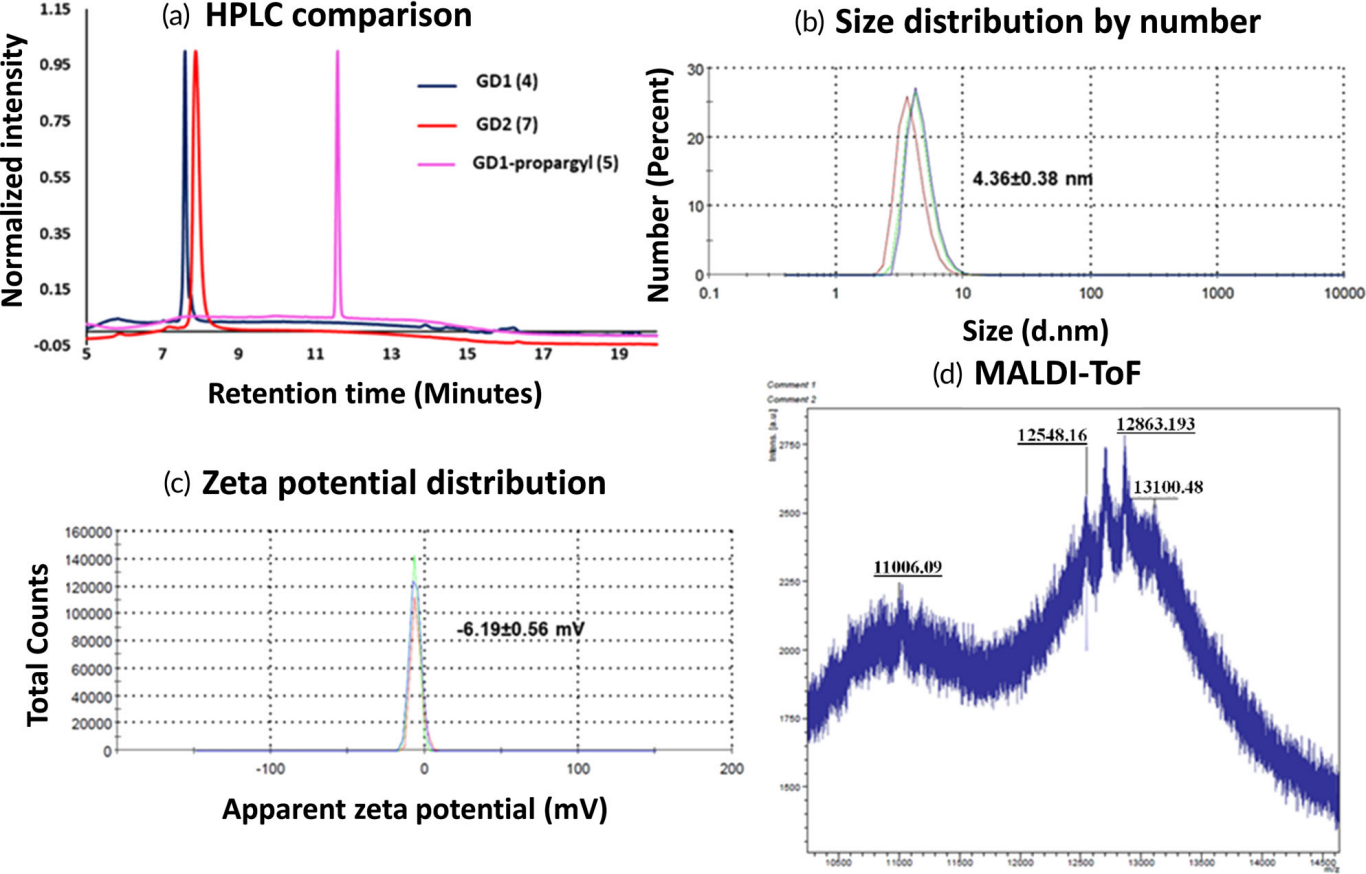
Physiochemical characterization of GD2. (a) HPLC traces of GD1, the propargyl derivative of GD1, and GD2; (b) size distribution; (c) zeta potential distribution via dynamic light scattering; and (d) MALDI‐TOF analysis of GD2.

The synthesis of glucose dendrimer (GD) began with the construction of hexapropargylated core (**1**) and AB4 peracetylated β‐D‐glucose‐PEG4‐azide building blocks using previously published literature procedures.^29^ To construct the dendrimer, the copper‐catalyzed alkyne azide cycloaddition (CuAAC) click reaction was performed between the hexa‐propargylated core (**1**) and the peracetylated β‐glucose‐PEG4‐azide (**2**) using classical click reagents, a catalytic amount of copper sulfate pentahydrate and sodium ascorbate to achieve compound **3** (Figure 1). The formation of the compound **3** was confirmed using ^1^H NMR where the propargyl peak in the core at *δ* 2.4 ppm disappeared and a new triazole peak appeared at *δ* 7.7 ppm confirmed the successful completion of click reaction (Figure 2). The treatment of peracetylated dendrimer (**3**) under Zemplén conditions provided generation 1 glucose dendrimer (GD1; **4**). Complete acetate deprotection was confirmed by the absence of acetate protons **δ** 2.0 ppm in ^1^H NMR spectrum (Figure 2). The terminal OH groups in GD1 dendrimer **4** were then modified using NaH and propargyl bromide to obtain GD1‐propargyl (**5**). The successful propargylation was confirmed by the appearance of alkyne peak at *δ* 2.4 ppm in NMR (Figure 2). The terminal alkyne groups in compound **5** were then reacted with β‐glucose‐PEG4‐azide (**6**) to produce generation two glucose dendrimer GD2 (**7**). The completion of click reaction was confirmed by the absence of acetylene peak protons at *δ* 2.5 ppm and the presence of new triazole peaks at *δ* 8.0–7.8 ppm in ^1^H NMR spectrum (Figure 2). All the intermediates and final dendrimer were additionally characterized via Mass and HPLC (Appendix S1). The HPLC purity of GD2 is >99% (Figure 3a). The size and zeta potential of GD2 were measured using dynamic light scattering (DLS). GD2 demonstrated a size of ~4.4 nm with nearly neutral zeta potential of ~−6 mV (Figure 3b,c). The MALDI‐TOF spectrum was in agreement with the theoretical molecular weight of the GD2 (Figure 3d).

To evaluate the targeting capability of GD2 via confocal microscopy and fluorescence spectroscopy, we attached a near infra‐red dye cyanine 5 (Cy5) at its surface. We modified 2–3 OH groups on the surface of GD2 (**7**) by reacting with propargyl bromide in the presence of sodium hydride to get compound **8** (Figure 1) which was further reacted with Cy5‐azide using CuAAC click reaction to obtain fluorescently labeled GD2‐Cy5 (**9**).

### 3.2 Glucose dendrimers show selective neuronal uptake in primary neuronal cultures and ex vivo brain slices, whereas hydroxyl PAMAM dendrimers do not

**FIGURE 4:**
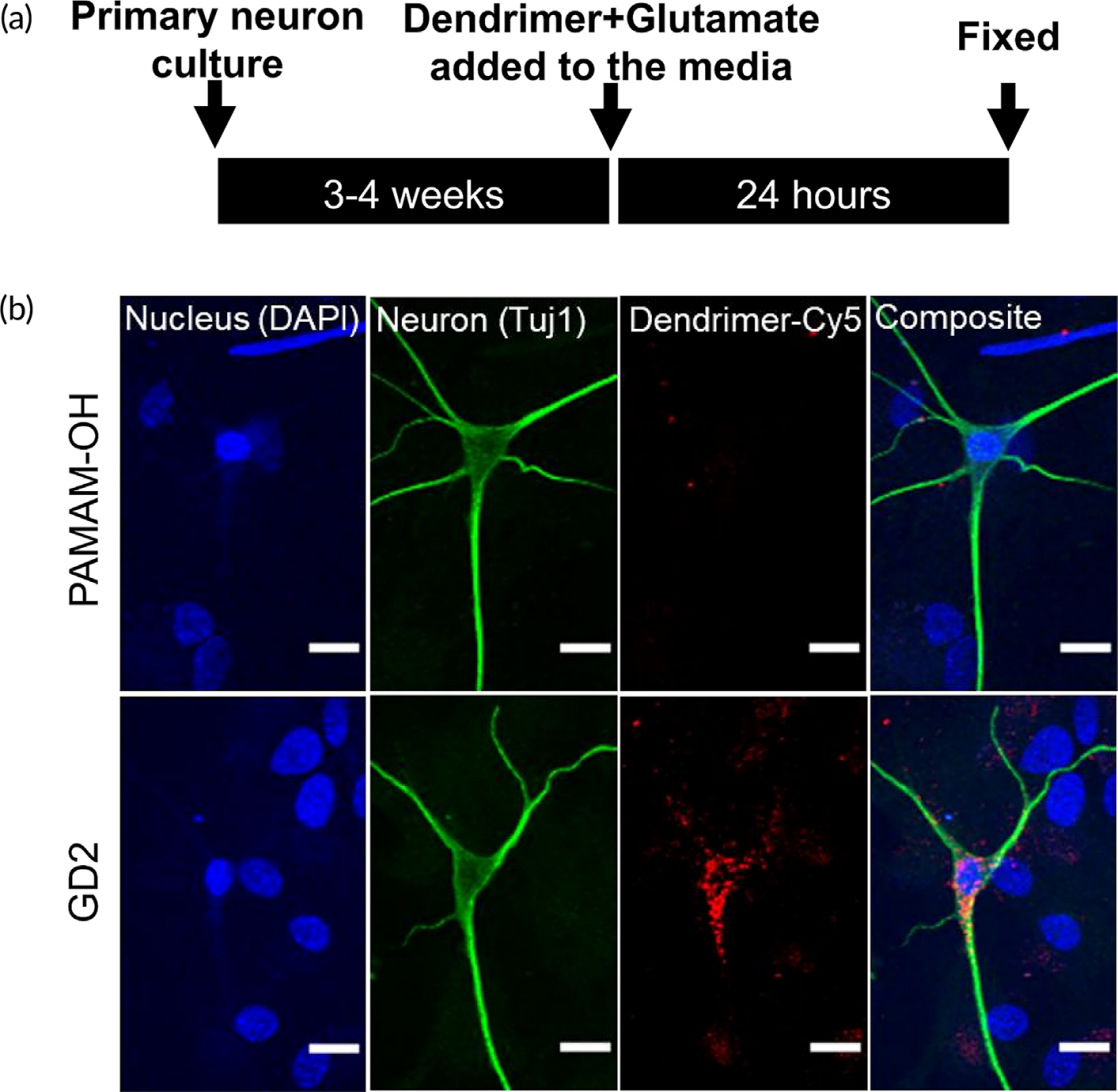
Intra‐neuronal uptake of GD2 in in vitro glutamate injury model. (a) Schema depicting the experimental timeline. Three to 4 weeks old primary neuronal cultures were incubated with 10 μM glutamate and 10 μg/mL dendrimer‐Cy5 for 24 h. Subsequently, the cultures were immuno‐stained with anti‐tubulin antibody to identify neurons. Confocal images were acquired and analyzed for the presence of dendrimer in Cy5‐channel (633–666 nm). (b) GD2 localized in pyramidal neurons whereas PAMAM‐OH did not. Scale bar: 20 μm.

Glutamate excitotoxicity is ubiquitous across many pathological brain conditions resulting in neuronal hyperexcitability and seizures.^38^ To mimic similar injury conditions, we cultured primary rabbit neuron cells and exposed it to 10 μM glutamate for 24 h that led to excitotoxicity of neurons.^39^ Concomitant exposure to 10 μg/mL glucose dendrimer for 24 h showed significant accumulation of GD2 in neurons. Primary neurons exposed to 10 μM glutamate and 10 μg/mL PAMAM‐OH had negligible accumulation of hydroxyl PAMAM‐OH dendrimer in neurons in this in vitro glutamate‐injury model (Figure 4).

**FIGURE 5:**
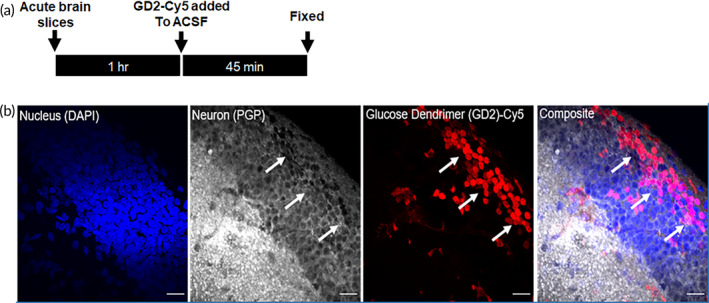
GD2 targets select CA1 neurons in acute hippocampal brain slices collected from rabbit model of cerebral palsy. (a) Experimental timeline showing the recovery time of 1 h after acute live brain slices followed by GD2‐Cy5 incubation in standard ACSF buffer for 45 min and subsequent formalin fixation. (b) Significant levels of GD2‐Cy5 were observed in CA1 pyramidal cell layer when hippocampal sections were incubated with GD2‐Cy5 (20 μg/mL) for 45 min in standard artificial cerebrospinal fluid (ACSF). Sections were washed and fixed with 10% formalin overnight followed by immuno‐staining for neuron‐specific marker: ubiquitin carboxy‐terminal hydrolase L1 (PGP). Arrow marks show the CA1 pyramidal neuron layer with GD2‐Cy5 accumulation in select neurons. Scale bar: 20 μm.

We further evaluated the unique neuronal targeting propensity of GD2 in ex vivo acute brain slices from newborn rabbits with brain injury caused by maternal systemic LPS‐induced inflammation (rabbit model of cerebral palsy).^31^ This ex vivo brain slice model provides unique advantages over primary neuronal culture as it largely preserves the neuronal intrinsic and synaptic architecture. Acute hippocampal brain sections from newborn rabbits with cerebral palsy, were incubated with GD2‐Cy5 (20 μg/mL) for 45 min (Figure 5a). The treated acute brain sections were formalin‐fixed and immune‐stained for neuronal markers: ubiquitin carboxy‐terminal hydrolase L1 (PGP) or MAP2. Confocal microscopy images showed abundance of GD2‐Cy5 in hippocampal pyramidal neurons (*n* = 3 rabbit kits), with minimal uptake in other cells and ‘healthy’ neurons in the vicinity (Figure 5). GD2 appears to localize in the cytoplasm and the nucleus of hyperexcitable neurons.

### 3.3 In vivo GD2‐Cy5 uptake in neurons depends on neuronal hyperexcitability

**FIGURE 6:**
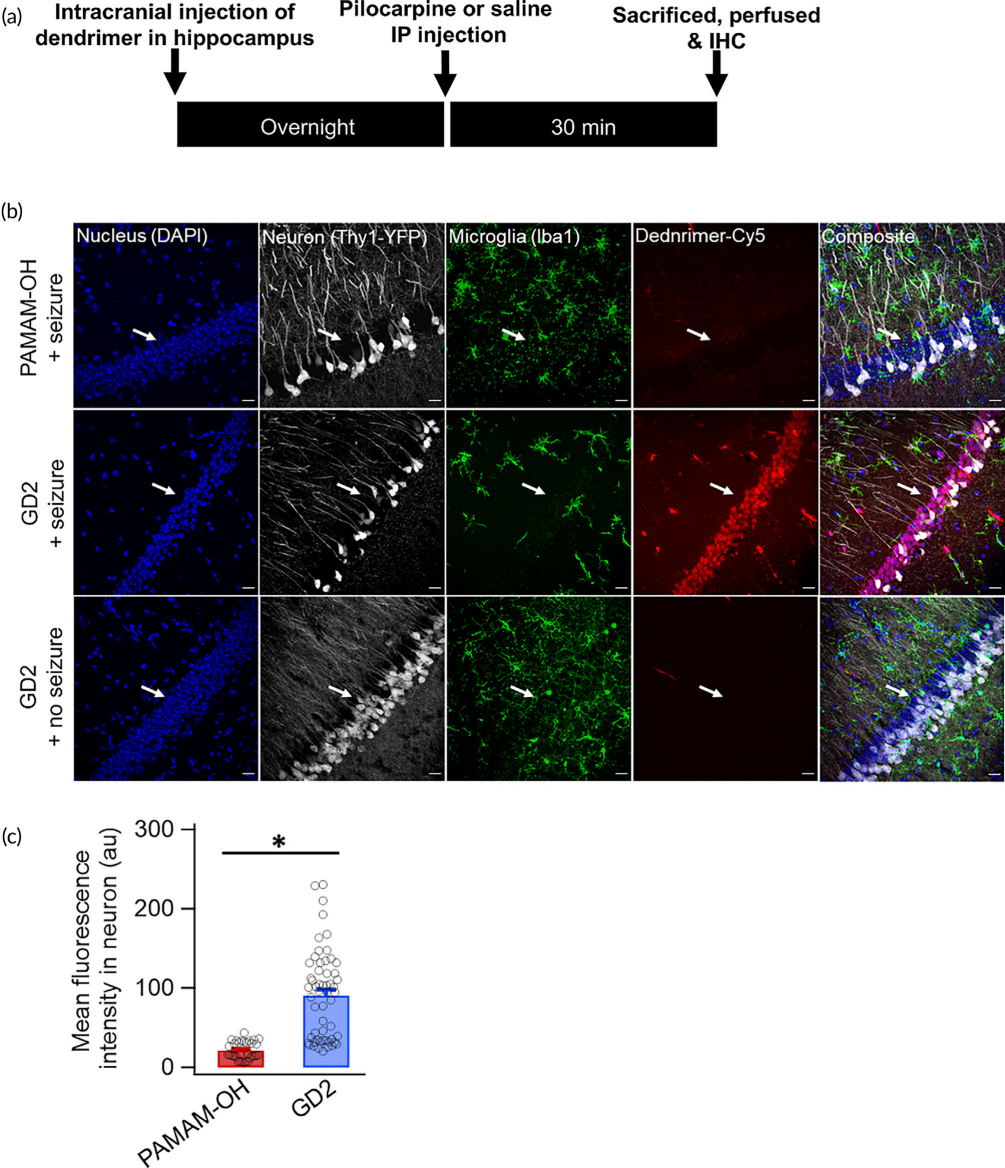
GD2 targets CA1 neurons in mouse model of seizures. (a) Schema depicting experimental timeline. PAMAM‐OH or GD2 conjugated to Cy5 was intra‐cranially administered in the right hemisphere. After overnight recovery, pilocarpine was injected to induce seizures. After 30 min of active behavioral seizures (Racine scale 3 or above), mice were sacrificed, perfused and brain collected for immunohistochemistry. (b) Representative confocal images from contralateral CA1 stained and imaged for DAPI (blue, nucleus), YFP‐expressing neurons (white), IBA1 (green, microglia), and dendrimer conjugated with Cy5 (red). White arrows indicate CA1 pyramidal neuron layer. Scale bar: 20 μm. (Top row): PAMAM‐OH uptake was negligible in the contralateral CA1 neurons. (Middle row): Marked increase in GD2 uptake by contralateral CA1 neurons upon seizure was evident. (Bottom row): in control mice without any seizure induction, contra‐lateral CA1 neurons did not show GD2 colocalization. (c) Mean dendrimer fluorescence intensity (Cy5 channel) from contralateral CA1 neurons show ~100 fold higher uptake of GD2 (*n* = 56 neurons from two mice) than PAMAM‐OH (*n* = 32 neurons from two mice). *p < 0.001.

Intracranial injection of PAMAM‐OH or GD2 conjugated to Cy5 (4 μL of 50 μg/μL) followed by seizure induction with 300 mg/kg of pilocarpine IP after 24 h, facilitated GD2 uptake by contralateral CA1 neurons. CA1 pyramidal neurons have been shown to be more vulnerable and demonstrate burst activity upon exposure to pilocarpine.^40^, ^41^ PAMAM‐OH uptake was negligible in the contralateral CA1 neurons (Figure 6).

These data demonstrate the feasibility of specific delivery of drugs by the GD2‐dendrimer platform to hyperexcitable neurons, primarily in the cytoplasm and the nucleus, in contrast to the hydroxyl PAMAM dendrimers.

### 3.4 Mechanism of uptake: glucose transporters mediate GD2‐Cy5 uptake

**FIGURE 7:**
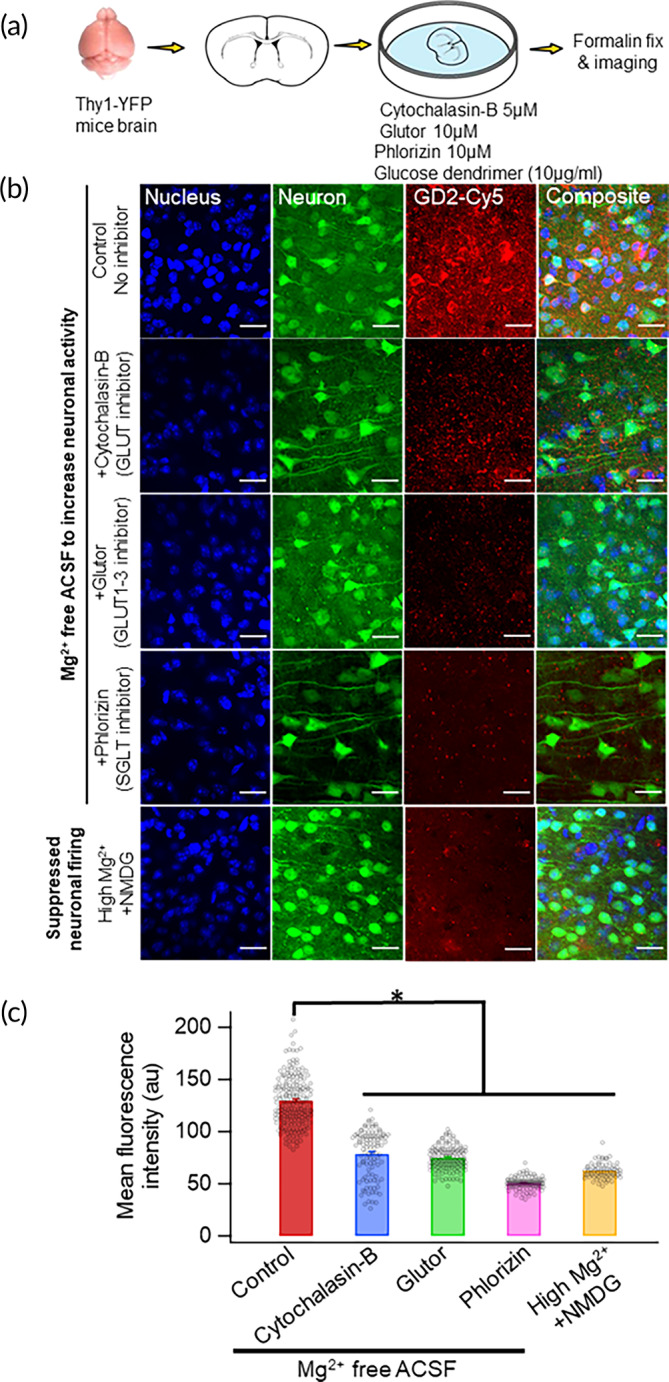
Neuronal activity and GLUT transporters mediate GD2 uptake. (a) 300 μm cortical brain sections were pre‐treated for 30 min with control ACSF (Mg^2+^ free, increases neuronal firing) or ACSF containing Mg^2+/^NMDG (suppressed neuronal activity) or control ACSF with either cytochalasin B (5 μM) or glutor (10 μM). After pre‐treatment, brain sections were incubated with GD2‐Cy5 (10 μg/mL) for 30 min followed by 10% formalin fixation and confocal imaging. Mean fluorescence intensities for GD‐Cy5 were evaluated from YFP‐expressing cortical neurons. (b) Confocal images of fixed sections showing DAPI (blue, nucleus), YFP (green, neurons), and GD2‐Cy5 (red) with different pharmacological treatments. Scale bar: 20 μm. (c) GD2‐Cy5 uptake was significantly decreased when neuronal activity was suppressed (*n* = 69 neurons), pre‐treated with cytochalasin B (*n* = 104 neurons) or glutor (*n* = 128 neurons) compared to control incubation condition (*n* = 187 neurons). *p < 0.001.

Higher metabolic activity by injured/hyperexcitable neurons can increase glucose requirement and thus can increase GD2 uptake. Accordingly, we hypothesized that suppression of neuronal activity will inhibit GD2 dendrimer uptake. Indeed, we found a significant decrease in GD2 colocalization in neurons when incubated with buffer solution containing *N*‐methyl‐D‐glucamine (instead of NaCl) and high MgCl_2_ (5 mM) known to suppress neuronal activity.^42^, ^43^ Furthermore, blocking glucose transporters (GLUT) using two different pharmacological antagonists [cytochalasin B (non‐specific GLUT inhibition) and glutor (GLUT 1–3 inhibitor)] diminished GD2 uptake by neurons, suggesting involvement of GLUT‐dependent uptake (Figure 7).

### 3.5 GD2 localizes in neurons upon intra‐nasal delivery in a mouse model of pilocarpine induced seizures

**FIGURE 8:**
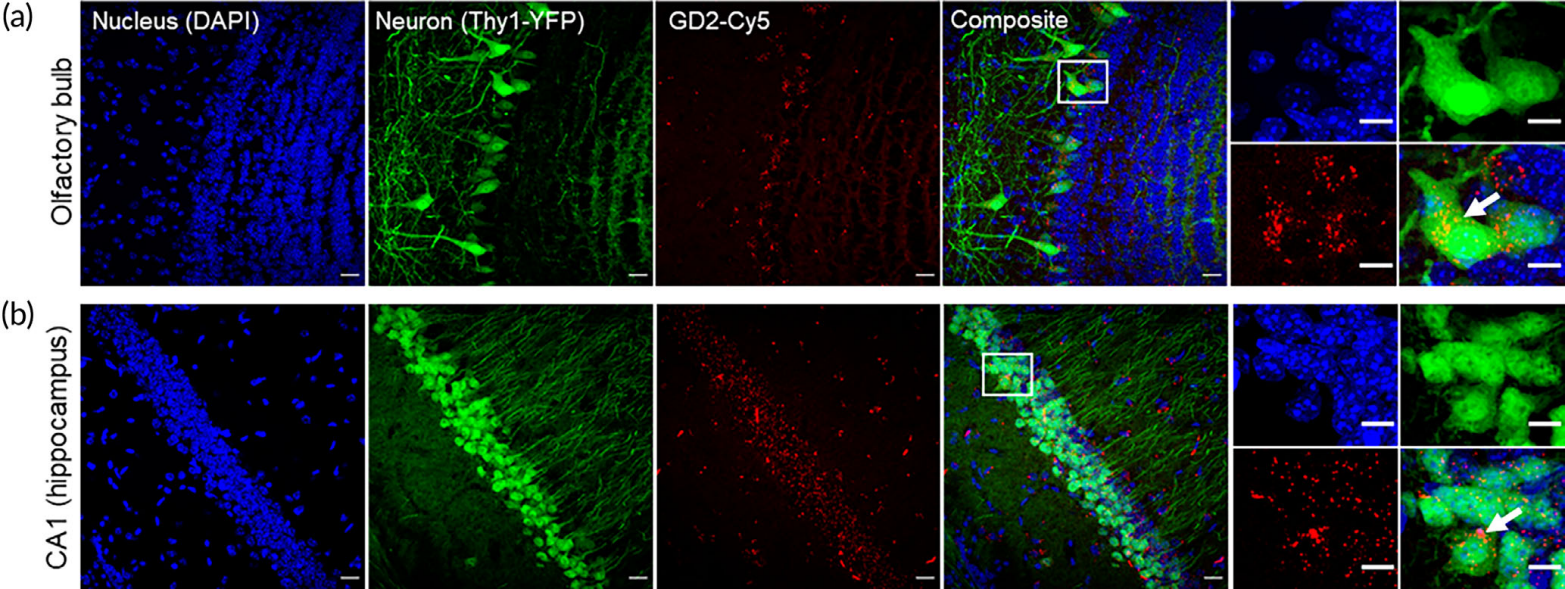
GD2‐Cy5 localizes in olfactory bulb and CA1 neuronal cell layer upon intra‐nasal administration. Ten microliters of GD2‐Cy5 (10 μg/μL in saline) was administered intra‐nasally during seizure and 4 h later, the brain was perfused and fixed. Confocal images of sections show lamellar Cy5 intensities in (a) olfactory bulb neuronal layer and (b) CA1 neuronal layer. DAPI (blue, nucleus), YFP (green, neurons), and GD2‐Cy5 (red). Scale bar: 20 μm. Representative higher magnification images are on the right‐most panels. Scale bar: 10 μm.

GD2‐Cy5 was administered intranasally (100 μg in 10 μL) following IP injection of 300 mg/kg of pilocarpine. Mice dosed with GD2‐Cy5 were perfused and fixed after 4 h. Confocal images show Cy5 intensities localized in neuronal layer both in olfactory bulb, cortex, and hippocampal CA1 region (Figure 8). This indicates that intranasal administration is a viable option for delivery of GD2 to the brain.

### 3.6 Synthesis and characterization of GD2‐valproic acid conjugate

**FIGURE 9:**
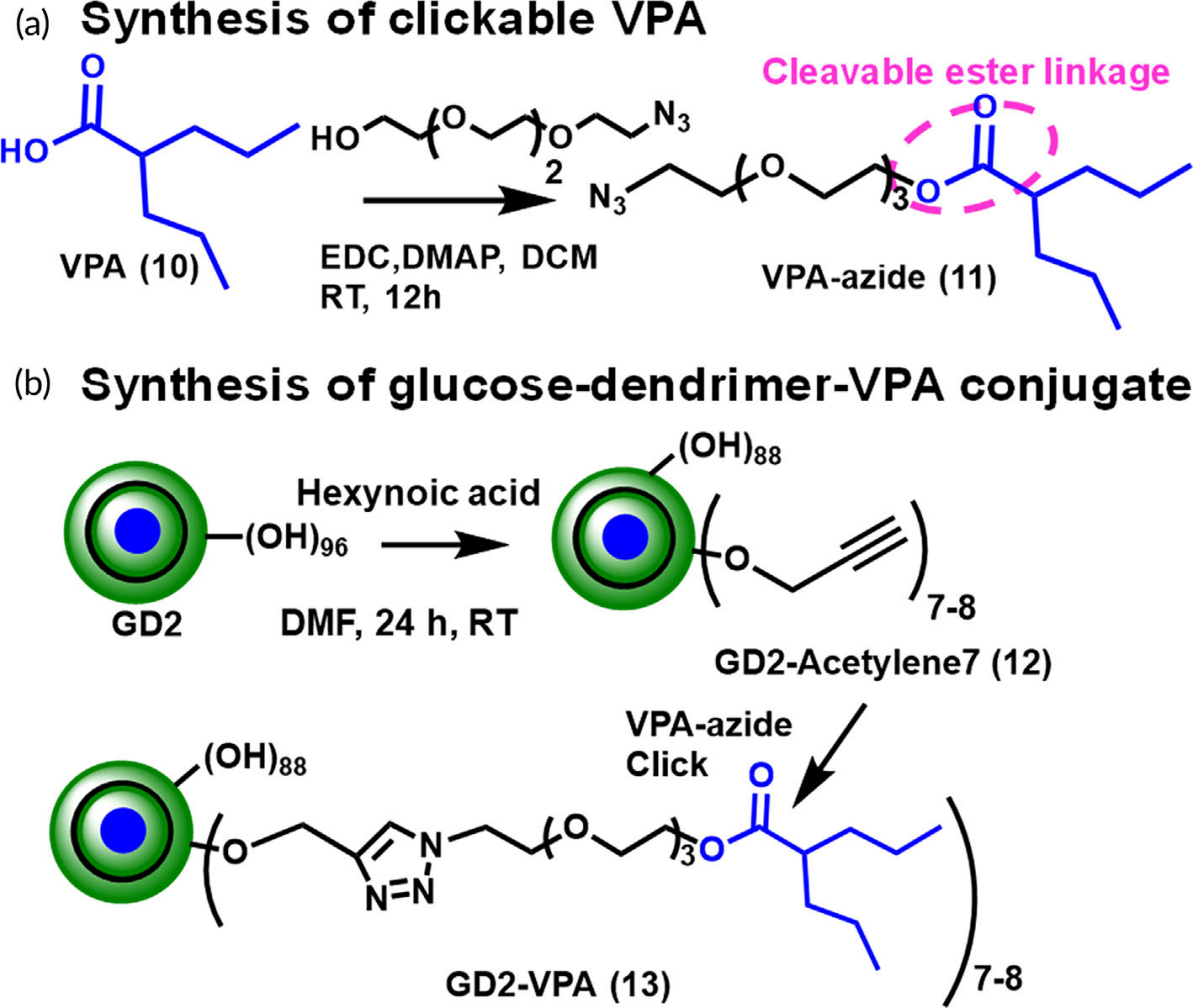
Synthetic route to GD2–VPA. (a) Synthesis of VPA‐azide with cleavable linker for intracellular release. (b) Synthesis of GD2–VPA via CuAAC click reaction.

**FIGURE 10:**
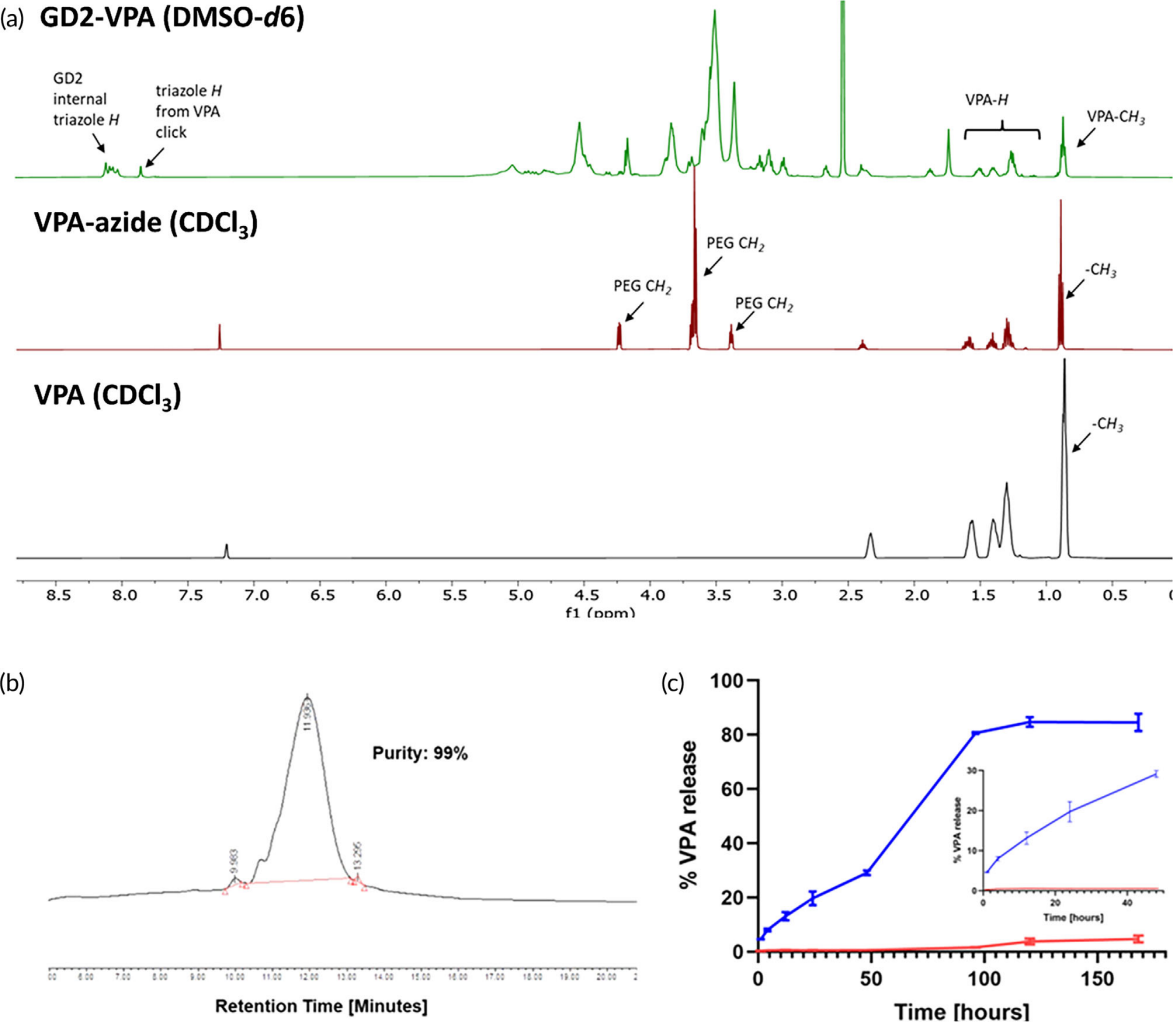
Characterization of GD2–VPA conjugate. (a) The figure represents the ^1^H NMR spectra of VPA (black), VPA‐azide (red), and GD2–VPA (green), clearly showing the characteristic protons corresponding to VPA and GD2. The appearance of a new triazole proton peak at *δ* 7.8 ppm confirms conjugate formation. (b) HPLC chromatograph of GD2–VPA at 210 nm (99% purity). (c) In vitro drug release profile of GD2–VPA showing the stability of the conjugate at plasma conditions (red) and the release of drug at intracellular conditions (blue).

Motivated by neuronal targeting by GD2‐Cy5, we conjugated valproate (VPA) to the GD2 dendrimer. The GD2–VPA conjugate was synthesized by first attaching an enzyme‐sensitive clickable linker on VPA (Figure 9a). The carboxylic acid group of VPA (**1**) was reacted with tetraethyleneglycol azide to obtain VPA‐azide (**2**). The ^1^H NMR clearly showed the presence of PEG protons at *δ* 3.4, 3.6, and 4.2 ppm (Figure 9a, red spectrum). On the other hand, GD2 (**3**) was partially modified by reacting ~7–8 hydroxyl groups with hexynoic acid in the presence of coupling agents to obtain an alkyne‐terminating GD2‐acetylene 7 (**12**) which was further reacted with VPA‐azide using Cu(I) catalyzed click (CuAAC) reaction in the presence of catalytic amount of CuSO_4_·5H_2_O and sodium ascorbate to obtain GD2–VPA (**13**) with on an average ~7–8 molecules of VPA attached on the surface of dendrimer (Figure 9b). The traces of copper were removed by dialyzing with ethylenediaminetetraacetic acid (EDTA). The final GD2–VPA conjugate was characterized by the NMR (Figure 10a) and HPLC (Figure 10b) and had an HPLC purity of 99%. The ^1^H NMR confirmed the product formation as evident by the presence of a triazole peak at δ 7.8 ppm (Figure 10a, green spectrum). The VPA loading on GD2–VPA conjugate is 6 wt% as calculated by the ^1^H NMR.

The GD–VPA was incubated at 37°C in sodium citrate buffer (pH 5.5) in the presence of esterase and phosphate‐buffered saline (PBS) buffer (pH 7.4). In the presence of esterase conditions at low pH which mimic lysosomal conditions, about 80% release of VPA from GD–VPA conjugate was observed by day 7.^44^, ^45^ While at PBS conditions, that mimic plasma conditions showed minimal VPA release up to 48 h and showed <6% VPA release by day 7 suggesting the plasma stability of the conjugate. This result provides indication of GD–VPA sustained drug release profile under the physiologically relevant, lysosomal conditions (Figure 10c).

### 3.7 GD2–VPA protects against pilocarpine‐induced behavioral seizures

**FIGURE 11:**
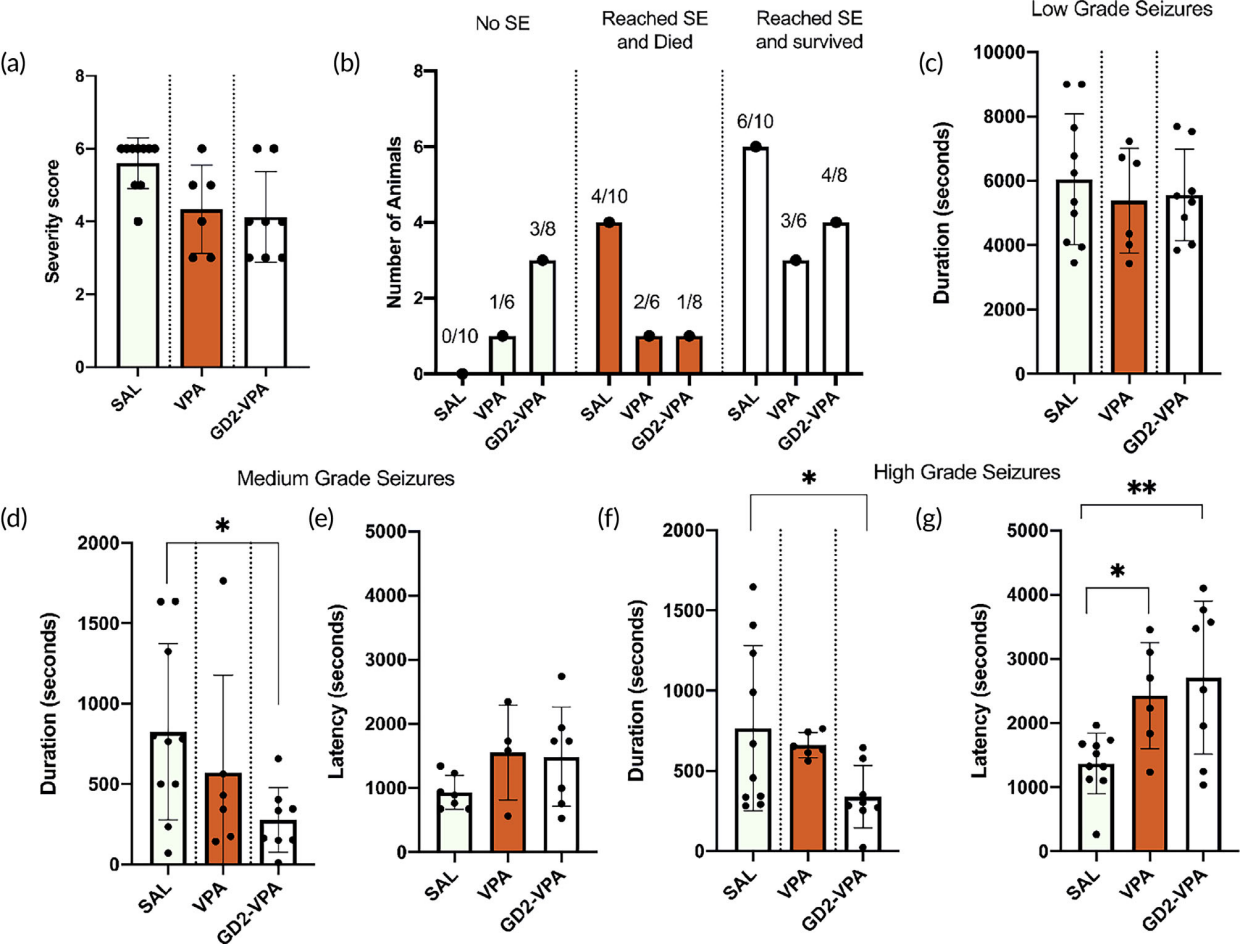
Intranasal GD2–VPA decreases pilocarpine induced seizure severity and duration. (a) Maximum severity score reached did not differ. (b) GD2–VPA increased the survival in the animals that reached status epilepticus (SE). GD2–VPA did not reduce the total duration of (c) low grade seizures but protected against the pilocarpine induced (d, e) medium grade seizures. (f) GD2–VPA also decreased the duration to high grade seizures and (g) increased the latency to first episode of high‐grade seizures post‐pilocarpine administration. **p < 0.01, *p < 0.05.

We evaluated the pharmacological efficacy of GD2–VPA using the pilocarpine model of behavioral seizures. There was an emergence of low‐grade seizures, described in stages 1 and 2, within 5 min of pilocarpine injection (300 mg/kg ip). We then administered saline, VPA or GD2–VPA intranasally (0.3 mg/kg on VPA basis) 15 min post‐pilocarpine injection. One to two microlitres of saline or GD2–VPA solution (10 μg/μL) was administered to each nostril every 2 min. Although there was no significant difference in the maximum seizure severity score reached between the different treatment groups, GD2–VPA treated animals had a lower mortality and lower propensity to go into status epilepticus (SE) as compared to saline and VPA treated ones (Figure 11a,b). In the clinical setting, as SE progresses, the seizure severity increases, and the anti‐seizure medicines typically fail to protect moderate and high‐grade seizures. Interestingly, GD2–VPA increased the latency to first episode of high‐grade seizures and reduced the total duration of both medium and high‐grade seizures post‐pilocarpine administration (Figure 11d–g). This indicates that GD2–VPA is most effective in controlling severe seizures that are typically the most harmful and often refractory treatment.

## 4 DISCUSSION

Developing dendritic structures that can intrinsically target neurons can open opportunities to address unmet needs. We synthesized a generation‐2 dendrimer made of glucose and ethylene glycol that presents 24 glucose moieties (with 96 hydroxyl groups) on the dendrimer surface, using click chemistry and a scalable approach. Using different models of increasing complexity, we demonstrate that GD2 internalizes in neurons under hyperexcitable conditions. Pharmacological blocker experiments suggest that glucose dendrimers localize primarily in neurons and the uptake appears to be mediated by glucose transporter. This selective neuronal localization is seen upon local or intranasal administration. Interestingly, the lack of neuronal targeting by hydroxyl PAMAM dendrimer that has a similar surface hydroxyl density (64 OH groups), suggests that the interaction of glucose on the surface of the GD2 dendrimer with hyperexcitable neurons is key to this targeting, and is mechanistically different from those of hydroxyl dendrimers.^46^, ^47^, ^48^ Previous development of neuron targeting inorganic nanoparticles were limited to surface interactions and not internalization.^49^ GD2, however, can be internalized into the cytoplasm, thus can be used to target intracellular organelles, receptors, and macromolecules. The selectivity of GD2 to target neurons only in the presence of brain injury and associated neuronal hyperexcitability, as in the pilocarpine‐induced seizure model demonstrated in Figure 6 and not in ‘normal‐firing’ neurons otherwise seen in healthy conditions provides the much needed selectivity for neurotherapeutics development. The importance of targeting drugs to metabolically active neurons in the context of epilepsy and seizures have been highlighted by many recent studies.^50^, ^51^ Moreover, treatment with intranasal GD2–VPA led to improvement in seizure frequency and mobility in the acute phase, indicating that this is a powerful platform to deliver drugs specifically to the neurons, which can reduce the side effects of free VPA in the clinic. This would also be applicable for other potent anti‐epileptic drugs many of which have debilitating side effects. Some prior studies have shown that glucose‐functionalization of gold nanoparticle–drug conjugates enhanced BBB transport, brain uptake in epilepsy models, improving efficacy of drugs, even though the specific neuronal uptake shown in this study is unique.^52^

Neurons maintain negative membrane potential at resting state and transiently depolarizes and repolarizes during an active action potential.^16^ Besides, transient membrane potential fluctuations also occur during sub‐threshold synaptic neuro‐transmission.^17^ Preserving the neuronal membrane polarization is an active process that requires cellular ATP as is required for action potential generation, ion concentration restoration, or vesicular recycling.^16^, ^17^ With neuronal stimulation, glucose can directly be transported intracellularly through glucose transporters^20^, ^21^, ^23^, ^53^ to meet the increased energy demands. Increased neuronal cellular uptake of glucose is facilitated through increased expression and translocation of Glut transporters following excitotoxic injury.^54^, ^55^, ^56^, ^57^ Increased activity and expression of neuronal glucose transporters can drive binding of GD2 to the transporter. Glut3 transporters are expressed in neuronal dendrites and axons.^58^ During acute brain injury, synaptic neurotransmission is increased that will necessitate higher synaptic vesicular recycling.^59^ It is also possible that GD2 proximity to neuronal membrane through Glut receptor interaction can lead to internalization of the dendrimer through synaptic vesicular recycling^60^, ^61^, ^62^ This may explain the preferential accumulation of GD2 in hyperexcitable neurons over other Glut expressing cells. The glucose dendrimer also demonstrates significant nuclear localization. Since VPA is known as an effective HDAC inhibitor, it is possible that the nuclear delivery of VPA by GD may also have long term neuroprotective effects which were not elicited in the timelines studied here. It is also possible that GD may be an effective platform for gene delivery to neurons.

Pilocarpine administration results in SE characterized by tonic–clinic generalized seizures. The initial seizures are triggered by the cholinergic activation of excitatory neurons, however the complex evolution of SE involves glutamatergic and GABAergic pathways.^63^ VPA is known to increase regional neuronal concentrations of GABA, an inhibitory neurotransmitter, by increasing its synthesis and altering GABA metabolism.^64^ An increase in GABA, upon GD2–VPA treatment can exert pronounced inhibitory effects to limit hyperexcitability. Additionally, the acute effects of GD2–VPA could also be mediated through blockade of voltage‐gated sodium channels^65^ suppressing excessive synchronized spiking. VPA might interact with the sodium channel's inactivation gate, affecting the channel's conformation, and the permeability to sodium ions.^66^ In GD2–VPA efficacy study, we observed decrement in seizure duration and latency primarily for high grade seizures, which implies that GD2–VPA is impeding mechanisms that can trigger global synchronization.^67^ Cumulative effects of GD2–VPA through multiple mechanisms can result in cessation of higher‐grade seizures. The low‐grade seizures may represent the pilocarpine‐mediated direct cholinergic activation induced residual seizure that could not be countered by GD2–VPA because of systemic pilocarpine presence. In summary, we have developed a dendrimer that intrinsically targets and localizes intracellularly in hyperexcitable neurons.

### 4.1 Potential for clinical translation and challenges

There are several unique advantages of this dendrimer platform. The glucose dendrimer is relatively easier to synthesize due to its modular building blocks and is scalable, enabling translation. It selectively targets hypermetabolic and injured neurons in the brain that can allow neuronal targeting of drugs using this platform. This selective targeting also enables a much lower dose to be effective which avoids systemic side effects that are commonly seen with VPA. Since the conjugate is expected to be cleared intact from off‐target organs, we expect that the drug side effects will be reduced. The intranasal route of administration for the management of acute seizures is also a strength since rapid delivery of the drug is possible. Further studies have to be done to better characterize different routes of administration, long term efficacy and the ability of GD–VPA to prevent delayed neuronal degeneration, before this therapy can be translated clinically. It is also possible that neurons that are in the later stages of cell death express less GLUT receptors and become metabolically hypoactive at which time point there could be decreased uptake of the GD–VPA. Optimal timing of administration may need to be further defined. It is also possible that other mechanisms of uptake may be involved in degenerating neurons. Future studies will focus on further correlating GD uptake in different types of neurons and at various stages of injury.

## 5 CONCLUSION

Preserving neurons and attenuating neuronal injury is the major goal for the treatment of many neurological disorders, which still remain as unmet needs due to our inability to target and deliver drugs to neurons. Utilizing the unique interaction of glucose and the function of GLUT transporters in hyperexcitable neurons, we have designed a dendrimer that intrinsically targets neurons under pathological conditions, even upon intranasal delivery, that is practical in indications such as seizures where rapid administration and uptake are critical. We show that it is not the surface hydroxyl density since a similar hydroxyl PAMAM dendrimer did not show this unique neuronal localization in the same models, suggesting an uptake mechanism of the dendrimer driven by surface moiety (glucose in this case), rather than surface groups (in the case of hydroxyl PAMAM dendrimers). It is the interaction of the glucose moiety presented on a dendrimer surface, and its interaction with the overexpressed GLUT receptors on the metabolically active neurons. The glucose dendrimer also shows nuclear localization that may have implications for other mechanisms of action of VPA. Since VPA is known as an effective HDAC inhibitor, it is possible that the nuclear delivery of VPA by GD may also have long term neuroprotective effects which were not elicited in the timelines studied here. It is also possible that GD may be an effective platform for gene delivery to neurons. We demonstrate the in vivo proof of concept efficacy in a pilocarpine induced mouse model of seizures, where intranasally‐delivered glucose dendrimer–valproate conjugate significantly decreases the seizure‐severity and duration of seizures. The unique neuronal uptake of glucose dendrimers and the associated efficacy in a clinically relevant model of seizures, opens new avenues for targeted delivery to neurons, and treatment of CNS disorders.

## AUTHOR CONTRIBUTIONS

**Anjali Sharma:** Conceptualization (equal); data curation (equal); writing – original draft (equal); writing – review and editing (equal). **Nirnath Sah:** Data curation (lead); writing – original draft (equal); writing – review and editing (equal). **Rishi Sharma:** Conceptualization (equal); data curation (equal); writing – original draft (equal); writing – review and editing (equal). **Preeti Vyas:** Data curation (equal); writing – original draft (equal); writing – review and editing (equal). **Wathsala Liyanage:** Data curation (equal); writing – original draft (equal); writing – review and editing (equal). **Sujatha Kannan:** Conceptualization (equal); funding acquisition (equal); supervision (equal); writing – original draft (equal); writing – review and editing (equal). **Rangaramanujam M. Kannan:** Conceptualization (equal); funding acquisition (equal); supervision (equal); writing – original draft (equal); writing – review and editing (equal).

## CONFLICT OF INTEREST STATEMENT

R. M. K., S. K., A. S., N. S., and R. S. are co‐inventors on patent applications relating to the compounds discussed here.

## Supporting information


**FIGURE S1.** ^13^C NMR of compound **3**.
**FIGURE S2.** MALDI‐TOF spectrum of compound **3**.
**FIGURE S3.** MALDI‐TOF spectrum of compound **4**.
**FIGURE S4.** HPLC trace of compound **4**.
**FIGURE S5.** MALDI‐TOF spectrum of compound **5**.
**FIGURE S6.** MALDI‐TOF spectrum of compound **7**.
**FIGURE S7.** HPLC trace of compound **7**.
**FIGURE S8.** ^1^H NMR of compound **9**.
**FIGURE S9.** HPLC trace of compound **9**.
**TABLE S1.** List of reagents used for in vivo and in vitro studies.

## Data Availability

The data are provided in the supporting information. Additional data are available on request.
