# Supplementary material for: Development of a novel glucose‐dendrimer based therapeutic targeting hyperexcitable neurons in neurological disorders
Source: Bioeng Transl Med. 2024 Mar 26;9(5):e10655. doi: 10.1002/btm2.10655 (PMC11561801; doi:10.1002/btm2.10655)
Supplement: Supplementary file 1 — FIGURE S1. 13C NMR of compound 3. FIGURE S2. MALDI‐TOF spectrum of compound 3. FIGURE S3. MALDI‐TOF spectrum of compound 4. FIGURE S4. HPLC trace of compound 4. FIGURE S5. MALDI‐TOF spectrum of compound 5. FIGURE S6. MALDI‐TOF spectrum of compound 7. FIGURE S7. HPLC trace of compound 7. FIGURE S8. 1H NMR of compound 9. FIGURE S9. HPLC trace of compound 9. TABLE S1. List of reagents used for in vivo and in vitro studies. [file BTM2-9-e10655-s001.docx]

**^Supporting Information^**

**^Development of a Novel Glucose-Dendrimer based Therapeutic Targeting Hyperexcitable Neurons in Neurological Disorders^**

Anjali Sharma^1,#,$^, Nirnath Sah^2,#^, Rishi Sharma^1,#,$^, Preeti Vyas^2^, Wathsala Liyanage^1^, Sujatha Kannan^2^,*, Rangaramanujam M. Kannan^1^,*

^1^Center for Nanomedicine at the Wilmer Eye Institute, Johns Hopkins University School of Medicine, Baltimore, MD, 21231, USA

^2^Anesthesiology and Critical Care Medicine, Johns Hopkins University School of Medicine, Baltimore, MD, 21287, USA

Short Title: Neuron targeting glucose-dendrimer

***Corresponding authors:**

Rangaramanujam M. Kannan, PhD

Mailing address: Center for Nanomedicine, Department of Ophthalmology, Wilmer Eye Institute, Johns Hopkins University School of Medicine, Baltimore, Maryland 21231,

Email: krangar1@jhmi.edu. Tel: +1 443-287-8634. Fax: +1 443-287-8635.

Sujatha Kannan, MD

Mailing address: Department of Anesthesiology and Critical Care Medicine,

Charlotte Bloomberg Children's Center 6318D,

1800 Orleans Street Baltimore, MD, 21287

^#^Equal contribution

^$^Current Address: Department of Chemistry, Washington State University, Pullman, WA

**Figure S1: ^13^C NMR of Compound 3**


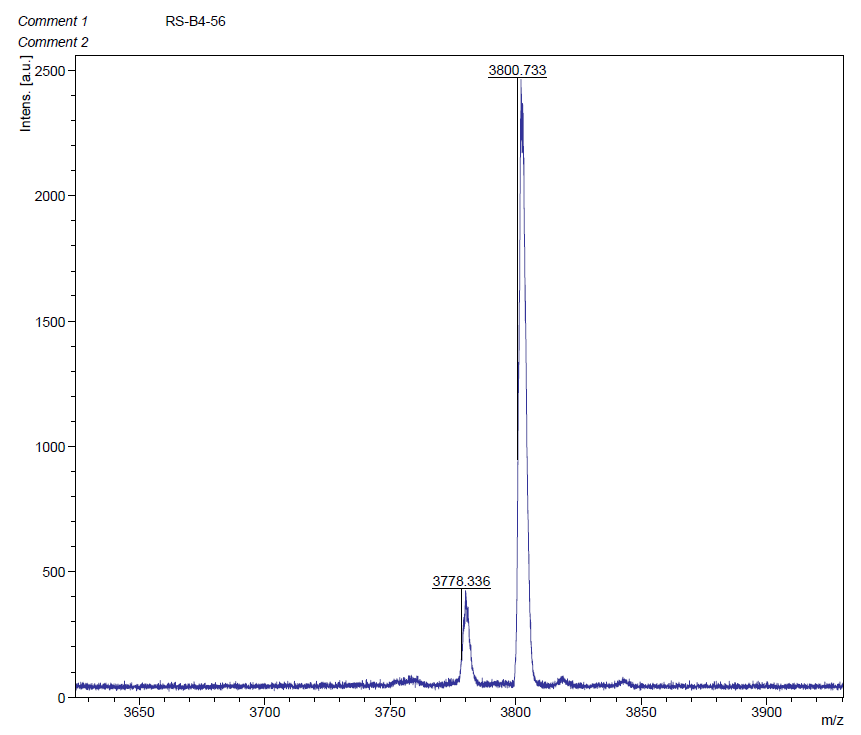


**Figure S2: MALDI-TOF spectrum of Compound 3**


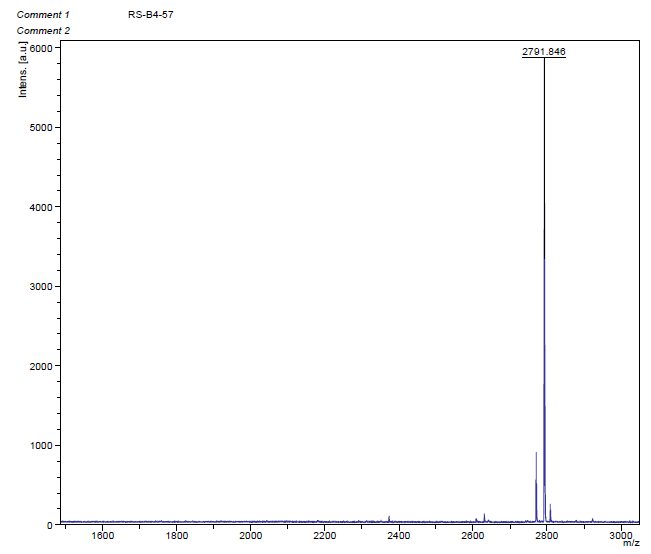


**Figure S3: MALDI-TOF spectrum of Compound 4**


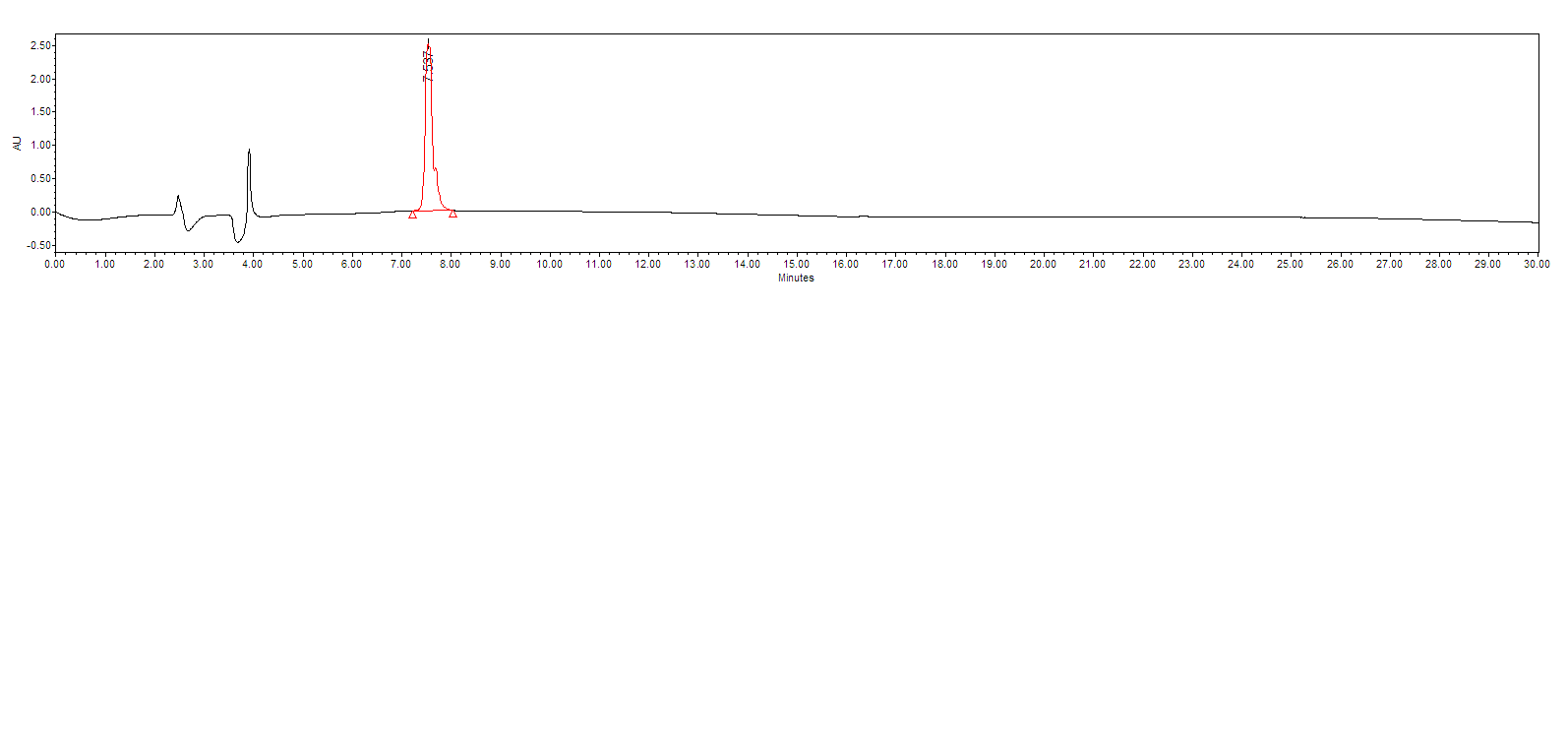


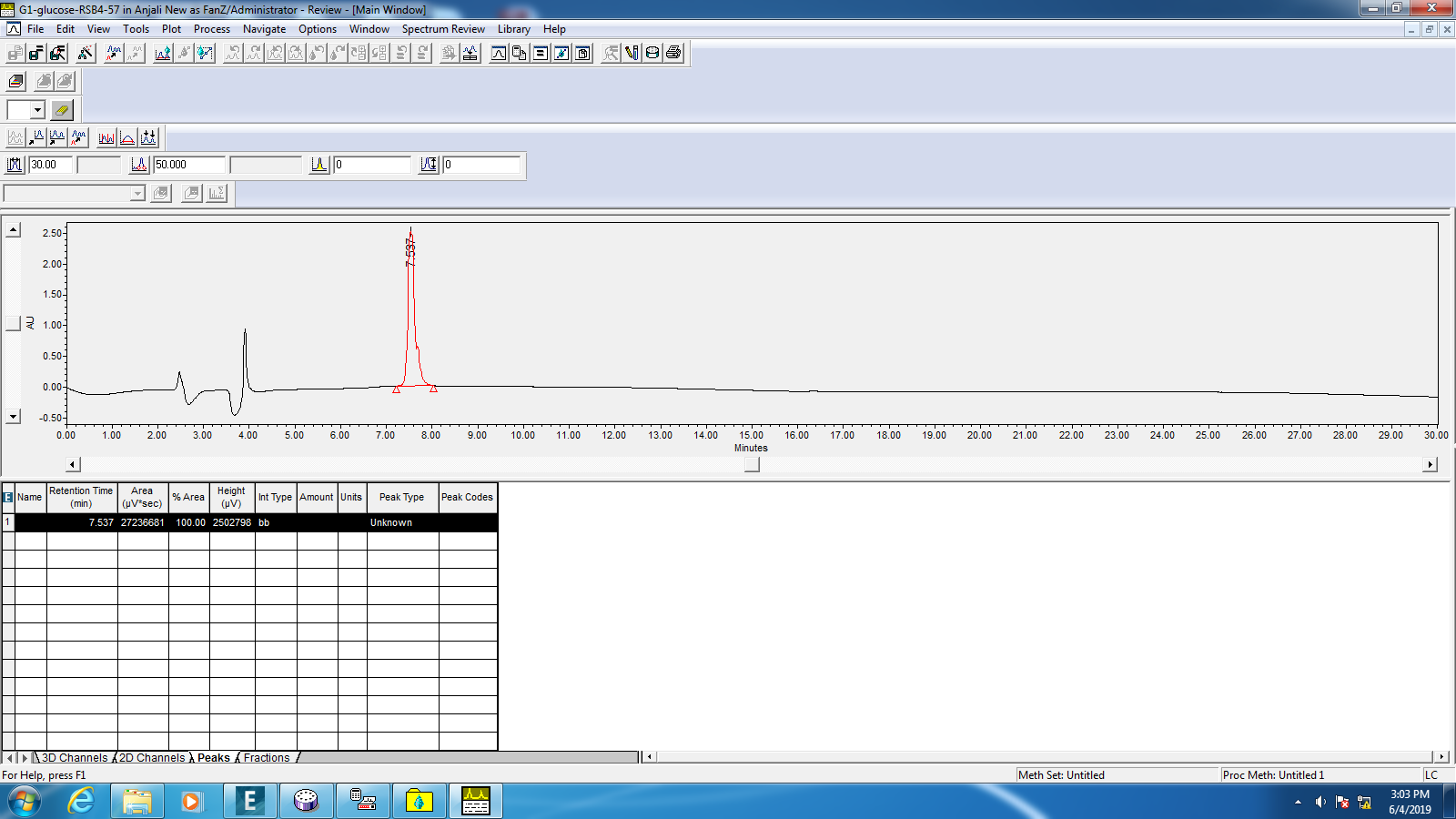


**Figure S4: HPLC trace of Compound 4**


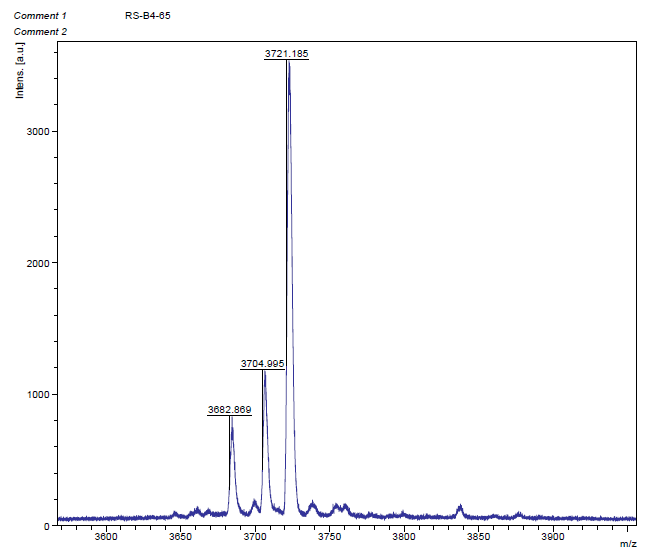


**Figure S5: MALDI-TOF spectrum of Compound 5**


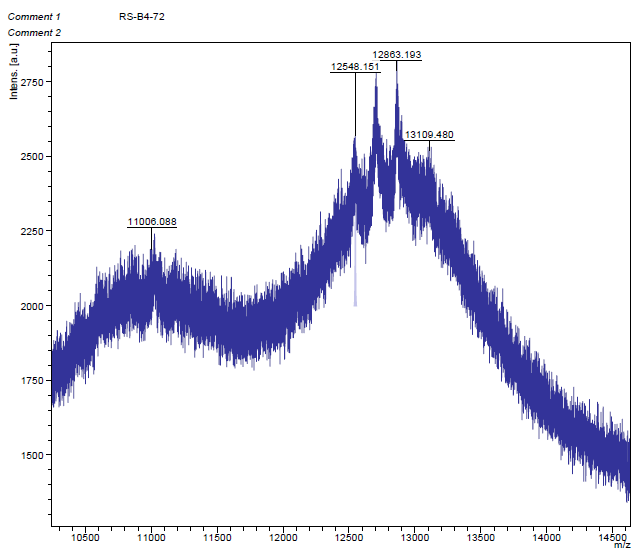


**Figure S6: MALDI-TOF spectrum of Compound 7**


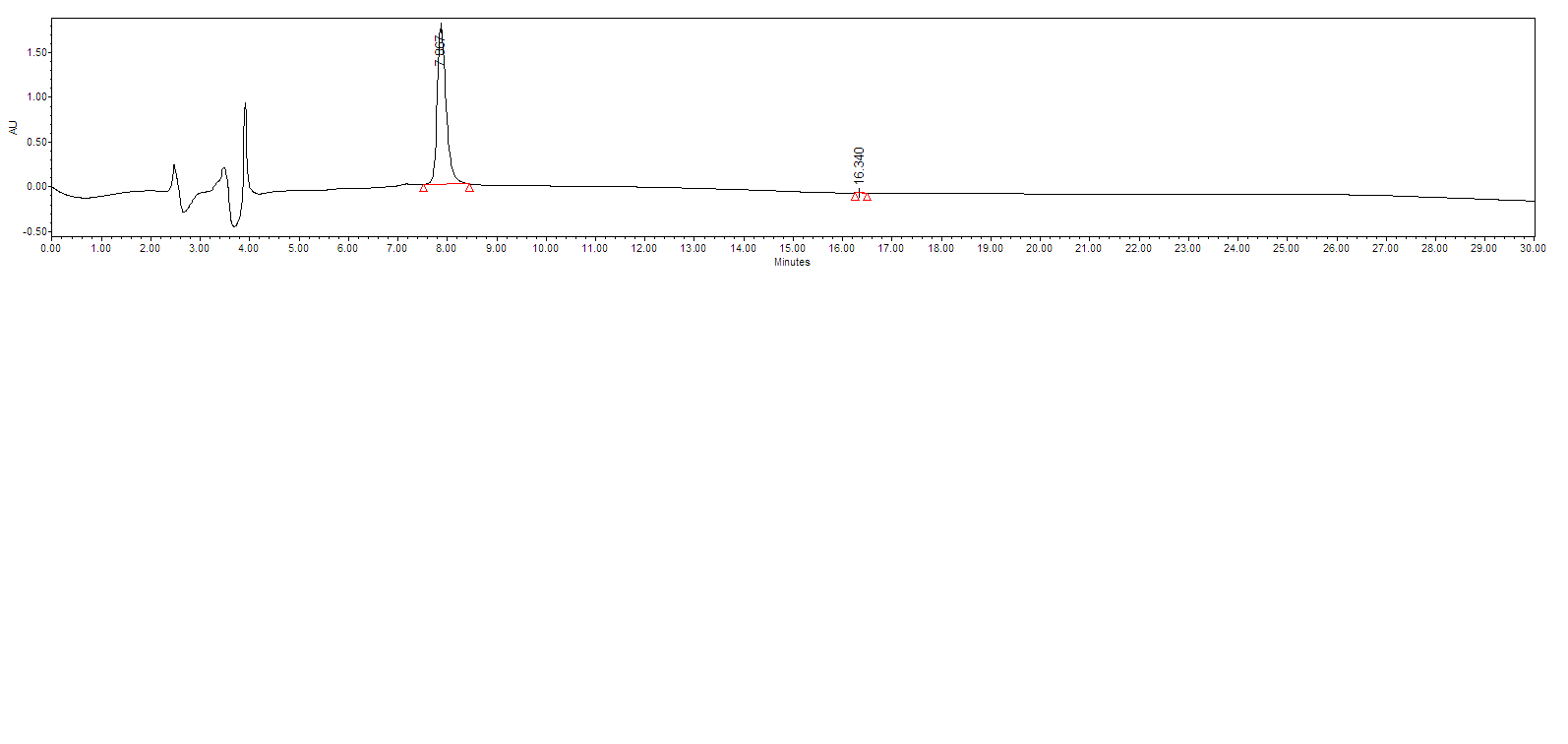


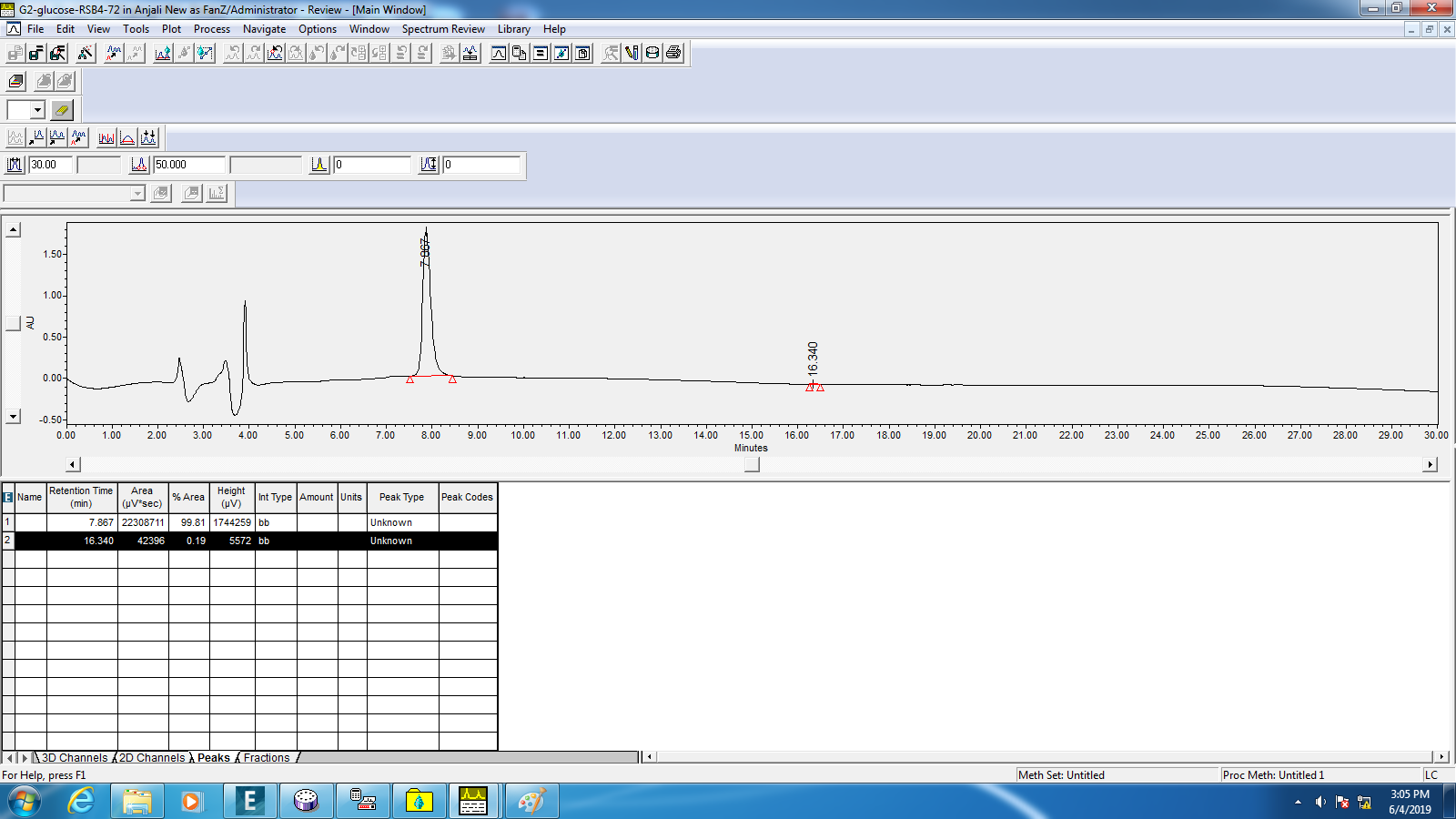


**Figure S7: HPLC trace of Compound 7**


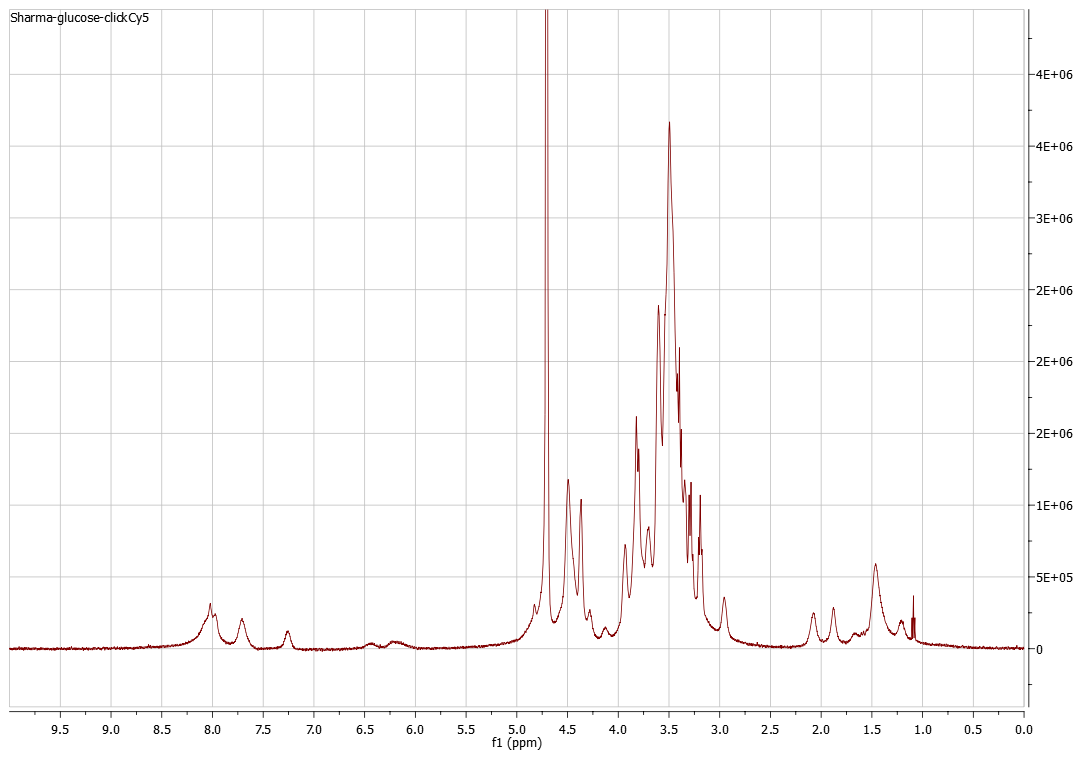


**Figure S8: ^1^H NMR of compound 9**


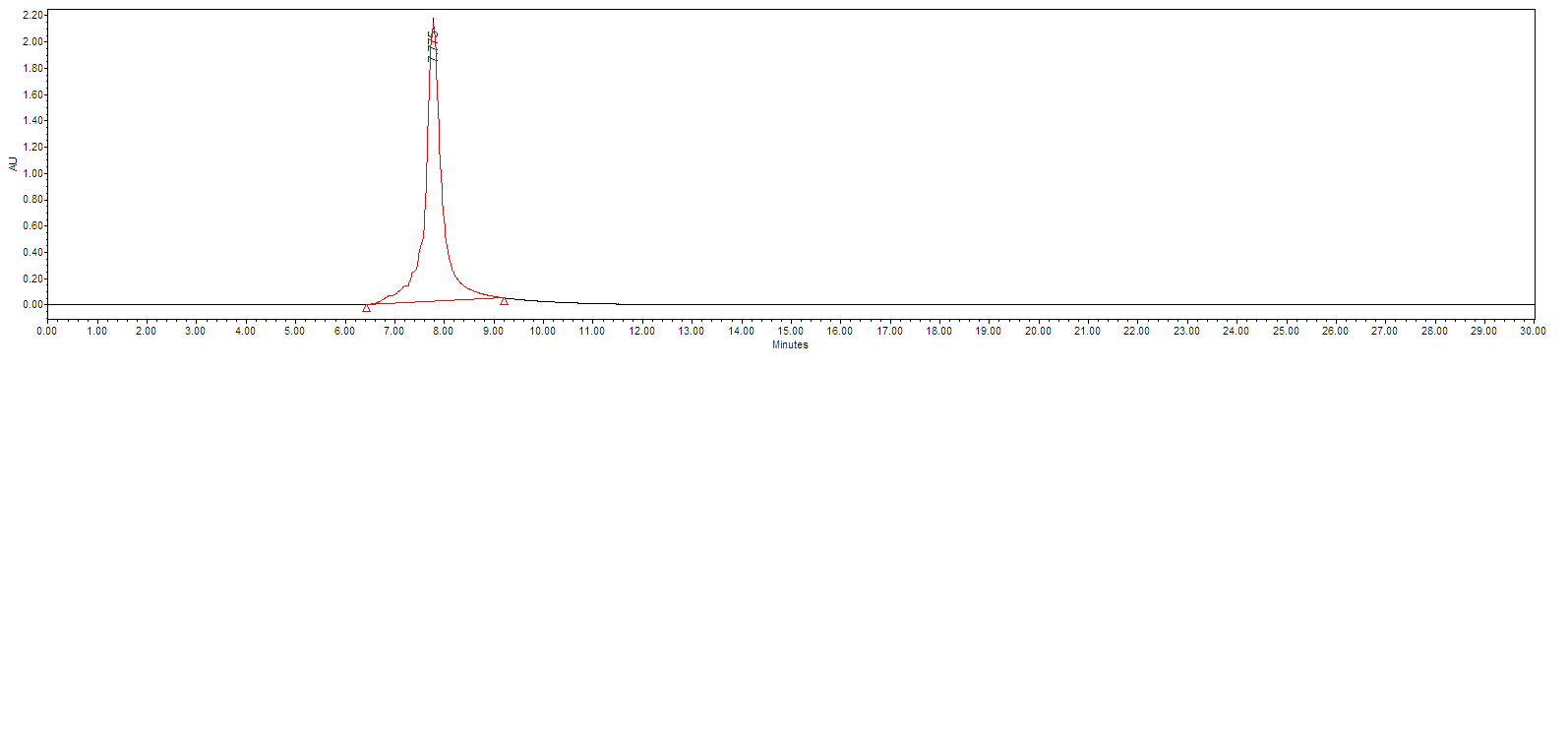


**Figure S9: HPLC trace of compound 9**

**Table1. List of reagents used for *in vivo* and *in vitro* studies:**

| **Reagent** | **Vendor** | **Catalogue no/RRID** |
| --- | --- | --- |
| Alexa fluor conjugated secondary antibody (donkey anti-chicken 488) | Jackson ImmunoResearch | RRID: AB_2340375 |
| Alexa fluor conjugated secondary antibody (donkey anti-goat 594) | Jackson ImmunoResearch | RRID: AB_2340432 |
| Alexa fluor conjugated secondary antibody (donkey anti-mouse 488) | Jackson ImmunoResearch | RRID: AB_2340846 |
| Alexa fluor conjugated secondary antibody (donkey anti-rabbit 488) | Jackson ImmunoResearch | RRID: AB_2313584 |
| AraC | Sigma-Aldrich | C6645-25 mg |
| B27 Supplements | Gibco, Thermo fischer scientific, USA | 17504044 |
| Calcium chloride | Sigma-Aldrich | C4901 (CAS: 10043-52-4) |
| Chicken anti-MAP2 antibody | Aves Lab, USA | MAP-0020 |
| D-Glucose | Sigma-Aldrich, USA | G7201 (CAS: 50-99-7) |
| DAPI | Invitrogen, USA | D1306 |
| Formalin | Cancer diagnostics, Inc | FX1000 |
| Glutamax | Gibco, Thermo fischer scientific, USA | 35050061 |
| Goat anti-IBA1 | Abcam, USA | RRID: AB_2224402 |
| HBSS (w/o Ca and Mg) | Gibco, Thermo fischer scientific, USA | 14185-052 |
| Hepes | Gibco, Thermo fischer scientific, USA | 15630080 |
| Horse Serum, heat inactivated | Gibco, Thermo fischer scientific, USA | 26050070 |
| LPS (Escherichia coli serotype O127:B8) | Sigma-Aldrich, USA | L3129 |
| Magnesium sulphate | Sigma-Aldrich, USA | M7506 (CAS: [7487-88-9](https://www.sigmaaldrich.com/US/en/search/7487-88-9?focus=products&page=1&perpage=30&sort=relevance&term=7487-88-9&type=cas_number)) |
| Mouse anti-PGP antibody | Abcam, USA | RRID: AB_306343 |
| Neurobasal Media | Gibco, Thermo fischer scientific, USA | 21103-049 |
| Papain Dissociation System without EBSS | Worthington, USA | LK003160 |
| Pen/Strep | Gibco, Thermo fischer scientific, USA | 1514012 |
| Pilocarpine hydrochloride | Sigma-Aldrich, USA | P650 |
| Pitocin (Oxytocin Injection, USP) | JHP Pharmaceuticals, Rochester, MI | NDC 42023-116-02 |
| Potassium Chloride | Sigma-Aldrich, USA | P3911 (CAS: [7447-40-7](https://www.sigmaaldrich.com/US/en/search/7447-40-7?focus=products&page=1&perpage=30&sort=relevance&term=7447-40-7&type=cas_number)) |
| Pyruvate | Gibco, Thermo fischer scientific, USA | 11360070 |
| Rabbit anti-beta III tubulin antibody | Abcam, USA | RRID: AB_444319 |
| Scopolamine methyl nitrate | Sigma-Aldrich, USA | S2250 |
| Sodium Bicarbonate | Sigma-Aldrich, USA | S6014 (CAS: 144-55-8) |
| Sodium Chloride | Sigma-Aldrich, USA | S9888 (CAS: [7647-14-5](https://www.sigmaaldrich.com/US/en/search/7647-14-5?focus=products&page=1&perpage=30&sort=relevance&term=7647-14-5&type=cas_number)) |
| Sodium Phosphate | Sigma-Aldrich, USA | S9763 (CAS: [7558-79-4](https://www.sigmaaldrich.com/US/en/search/7558-79-4?focus=products&page=1&perpage=30&sort=relevance&term=7558-79-4&type=cas_number)) |
| Sucrose | Sigma-Aldrich, USA | GC57501 |
